# Gene Expression Profile of *Bombyx mori* Hemocyte under the Stress of Destruxin A

**DOI:** 10.1371/journal.pone.0096170

**Published:** 2014-05-06

**Authors:** Liang Gong, Xiurun Chen, Chenglan Liu, Fengliang Jin, Qiongbo Hu

**Affiliations:** College of Natural Resource and Environment, South China Agricultural University, Guangzhou, China; Uppsala University, Sweden

## Abstract

Destruxin A (DA) is a cyclo-peptidic mycotoxin from the entomopathogenic fungus *Metarhizium anisopliae*. To uncover potential genes associated with its molecular mechanisms, a digital gene expression (DGE) profiling analysis was used to compare differentially expressed genes in the hemocytes of silkworm larvae treated with DA. Ten DGE libraries were constructed, sequenced, and assembled, and the unigenes with least 2.0-fold difference were further analyzed. The numbers of up-regulated genes were 10, 20, 18, 74 and 8, as well as the numbers of down-regulated genes were 0, 1, 8, 13 and 3 at 1, 4, 8, 12 and 24 h post treatment, respectively. Totally, the expression of 132 genes were significantly changed, among them, 1, 3 and 12 genes were continually up-regulated at 4, 3 and 2 different time points, respectively, while 1 gene was either up or down-regulated continually at 2 different time points. Furthermore, 68 genes were assigned to one or multiple gene ontology (GO) terms and 89 genes were assigned to specific Kyoto Encyclopedia of Genes and Genomes (KEGG) Orthology. In-depth analysis identified that these genes putatively involved in insecticide resistance, cell apoptosis, and innate immune defense. Finally, twenty differentially expressed genes were randomly chosen and validated by quantitative real-time PCR (qRT-PCR). Our studies provide insights into the toxic effect of this microbial insecticide on silkworm's hemocytes, and are helpful to better understanding of the molecular mechanisms of DA as a biological insecticide.

## Introduction

Entomopathogenic fungi, such as *Metarhizium anisopliae* and *Beauveria bassiana* [1], [2], are very important natural factors for insect control. As a result, fungal insecticides are very attractive in pests IPM (Integrated Pest Management), particularly in areas of high chemical insecticide resistance [3], [4]. The entomopathogenic fungus *M. anisopliae* has been well studied, and commercially used to control termites [5], grasshoppers [6], thrips [7], whiteflies [8] and red spider mites [9]. Notably, Destruxins were firstly isolated from *M. anisopliae*, and they are important virulence factors which accelerate the death of infected insects [4], [10]–[11]. However, the molecular mechanisms of destruxins as insecticide have not been elucidated yet.

Destruxins are fungal secondary metabolites and bio-synthesized by non-ribosomal peptide synthases [12]. Chemically, destruxins are cyclic hexadepsipeptides composed of an α-hydroxy acid and five amino acid residues. So far, 39 destruxins analogues have been isolated from *M. anisopliae* and other fungal species [13]–[15]. The common analogues, Destruxin A (DA), Destruxins B (DB) and Destruxins (DE) exhibit a substantial insecticidal activities against many species of pests such as *Plutella xylostella*, *Spodoptera litura*, *Manduca sexta* and *Pieris brassicae* [16]–[18], etc.

There is limited information regarding the mechanisms of action of destruxins. DA suppresses the contractions of visceral muscles of *Locusta migratoria*, with an influx of extracellular Ca^2+^ [19]. Additionally, DA strongly inhibits the secretion rate of *Rhodnius prolixus* Malpighian tubules fluid without infecting on the production of intracellular ATP [18]. Other studies demonstrated that DB selectively inhibited the activity of V-type ATPase from different cells [20]–[21]. Some experiments have shown that destruxins are able to damage the innate immunity of insects. The function of the encapsulation and phagocytosis processes of insect hemocytes were found to be destroyed by destruxins [22]. In our previously survey, it was also found that DA could induce obviously morphologic alterations of silkworm's hemocytes *in vivo*, even at an extremely low dose [23]. Furthermore, another study showed that destruxinss may play a key role in suppressing the innate immune response of *Drosophila melanogaster*, by inhibiting the expression of antimicrobial peptides [24]. However, more studies are needed for better understanding of the host immunity response modulated by destruxins, which may be an important aspect of the mechanisms of destruxins acting on insects.

Over the past several years, next-generation sequencing (NGS) techniques, such as Illumina/Solexa, have been developed as high-throughput and low-cost sequencing platforms to investigate the *de novo* transcriptome, gene expression profiling, and detecting methylation patterns [25], [26]. In the present study, a digital gene expression (DGE) profiling analysis using Illumina sequencing technology was used to compare differentially expressed genes in the hemocytes of silkworm larvae with DA or mock treatment. Ten DGE libraries were constructed, sequenced, and assembled, and the unigenes with least 2.0-fold difference in expression were further assembled and analyzed. Finally, a number of the differentially expressed genes were confirmed by quantitative real-time PCR (qRT-PCR).

## Results

### Illumina sequencing and reads assembly

**Figure 1:**
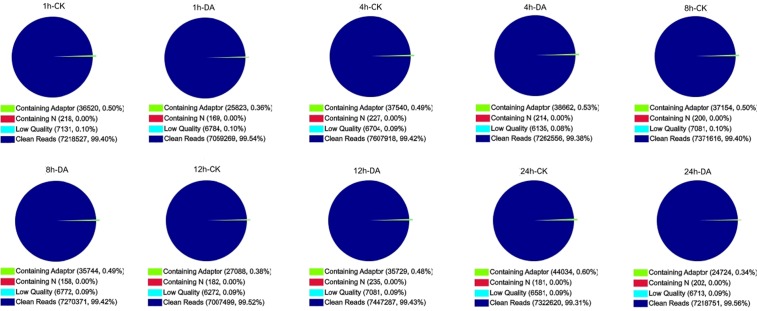
Quality assessment of reads in each library.

Ten DGE libraries of *B. mori* were sequenced including the DA-treated and control samples, which generated a number of row reads ranged from 7,041,039 to 7,652,389 for each of them. Following filtering the low quality reads, the total number of clean reads per library ranged from 7,007,499 to 7,607,918 million, and the percentage of clean reads in each library ranged from 99.31% to 99.56% (Figure 1). The alignment with reference transcriptome and reference genome showed that unique match ranged from 52.68% to 58.32%, and from 72.59% to 73.08%, respectively, which is the most critical subset of DGE libraries to identify a transcript precisely (Table 1). To evaluate if the number of detected genes increasing proportionally to total tags number, we performed the sequencing saturation analysis for each sample. The results showed that the number of detected genes was increasing as the number of reads was increasing. However, when the number of reads reached to 7.5 million, the growth rate of detected genes flattened, indicating that the number of detected genes tended to be saturated (Figure S1). We used the distribution of reads on the reference genes to evaluate the randomness. If the randomness is poor, reads preference to specific gene region will directly affect subsequent bioinformatics analysis. But our data (Figure S2) showed a even distribution with the dynamic range more than 9.560, which is a required ratio between the maximum and minimum expression level for RNA-Seq. To assess comparability of DGE data, we analyzed of the distributions of genes' coverage. The results are similar between the control and treated samples, indicating it is comparability (Figure S3).

**Table 1:** Quality assessment of reads in each library.

Sample ID	Total Reads	Total BasePairs	Total Mapped Reads	Perfect Match	< = 2 bp Mismatch	Uni)ue Match	Multi-position Match	Total UnmappeG Reads
mapping to reference genes								
1h-CK	7,218,527(100.00%)	353,707,823(100.00%)	4,338,614(60.10%)	3,303,737(45.77%)	1,034,877(14.34%)	4,203,040(58.23%)	135,574(1.88%)	2,879,913(39.90%)
1h-DA	7,059,269(100.00%)	345,904,181(100.00%)	3,976,792(56.33%)	3,026,423(42.87%)	950,369(13.46%)	3,846,164(54.48%)	130,628(1.85%)	3,082,477(43.67%)
4h-CK	7,607,918(100.00%)	372,787,982(100.00%)	4,208,364(55.32%)	3,210,421(42.20%)	997,943(13.12%)	4,062,080(53.39%)	146,284(1.92%)	3,399,554(44.68%)
4h-DA	7,262,556(100.00%)	355,865,244(100.00%)	3,972,549(54.70%)	3,025,332(41.66%)	947,217(13.04%)	3,825,954(52.68%)	146,595(2.02%)	3,290,007(45.30%)
8h-CK	7,371,616(100.00%)	361,209,184(100.00%)	4,119,567(55.88%)	3,128,946(42.45%)	990,621(13.44%)	3,984,631(54.05%)	134,936(1.83%)	3,252,049(44.12%)
8h-DA	7,270,371(100.00%)	356,248,179(100.00%)	4,069,945(55.98%)	3,071,129(42.24%)	998,816(13.74%)	3,918,331(53.89%)	151,614(2.09%)	3,200,426(44.02%)
12h-CK	7,007,499(100.00%)	343,367,451(100.00%)	4,024,328(57.43%)	3,087,657(44.06%)	936,671(13.37%)	3,879,756(55.37%)	144,572(2.06%)	2,983,171(42.57%)
12h-DA	7,447,287(100.00%)	364,917,063(100.00%)	4,387,447(58.91%)	3,332,790(44.75%)	1,054,657(14.16%)	4,226,722(56.76%)	160,725(2.16%)	3,059,840(41.09%)
24h-CK	7,322,620(100.00%)	358,808,380(100.00%)	4,381,281(59.83%)	3,351,541(45.77%)	1,029,740(14.06%)	4,231,254(57.78%)	150,027(2.05%)	2,941,339(40.17%)
24h-DA	7,218,751(100.00%)	353,718,799(100.00%)	4,388,131(60.79%)	3,327,038(46.09%)	1,061,093(14.70%)	4,209,788(58.32%)	178,343(2.47%)	2,830,620(39.21%)
mapping to reference genome								
1h-CK	7,218,527(100.00%)	353,707,823(100.00%)	5,753,578(79.71%)	4,188,473(58.02%)	1,565,105(21.68%)	5,258,986(72.85%)	494,592(6.85%)	1,464,949(20.29%)
1h-DA	7,059,269(100.00%)	345,904,181(100.00%)	5,633,325(79.80%)	4,095,419(58.01%)	1,537,906(21.79%)	5,137,525(72.78%)	495,800(7.02%)	1,425,944(20.20%)
4h-CK	7,607,918(100.00%)	372,787,982(100.00%)	6,092,604(80.08%)	4,449,564(58.49%)	1,643,040(21.60%)	5,538,986(72.81%)	553,618(7.28%)	1,515,314(19.92%)
4h-DA	7,262,556(100.00%)	355,865,244(100.00%)	5,814,310(80.06%)	4,235,398(58.32%)	1,578,912(21.74%)	5,277,498(72.67%)	536,812(7.39%)	1,448,246(19.94%)
8h-CK	7,371,616(100.00%)	361,209,184(100.00%)	5,914,326(80.23%)	4,293,595(58.24%)	1,620,731(21.99%)	5,367,432(72.81%)	546,894(7.42%)	1,457,290(19.77%)
8h-DA	7,270,371(100.00%)	356,248,179(100.00%)	5,815,639(79.99%)	4,200,774(57.78%)	1,614,865(22.21%)	5,260,111(72.35%)	555,528(7.64%)	1,454,732(20.01%)
12h-CK	7,007,499(100.00%)	343,367,451(100.00%)	5,643,292(80.53%)	4,145,460(59.16%)	1,497,832(21.37%)	5,121,247(73.08%)	522,045(7.45%)	1,364,207(19.47%)
12h-DA	7,447,287(100.00%)	364,917,063(100.00%)	5,991,286(80.45%)	4,365,692(58.62%)	1,625,594(21.83%)	5,420,779(72.79%)	570,507(7.66%)	1,456,001(19.55%)
24h-CK	7,322,620(100.00%)	358,808,380(100.00%)	5,893,436(80.48%)	4,322,509(59.03%)	1,570,927(21.45%)	5,335,314(72.86%)	558,122(7.62%)	1,429,184(19.52%)
24h-DA	7,218,751(100.00%)	353,718,799(100.00%)	5,811,085(80.50%)	4,226,172(58.54%)	1,584,913(21.96%)	5,240,312(72.59%)	570,773(7.91%)	1,407,666(19.50%)

### Functional annotation of differentially expressed genes

**Figure 2:**
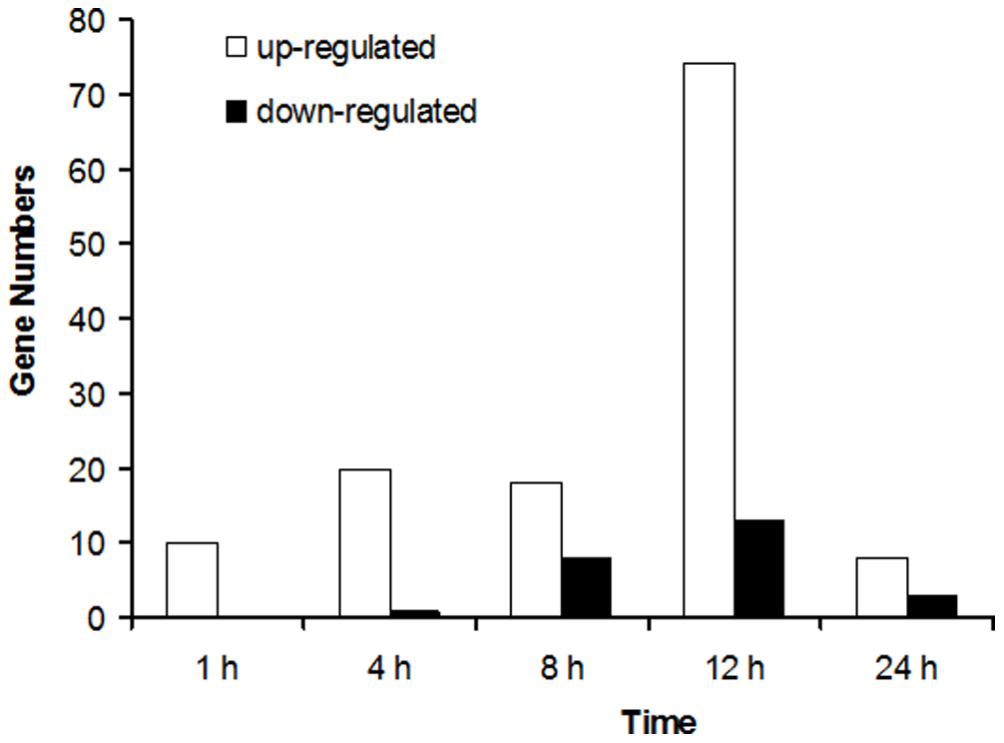
Screening of more than 2-fold differentially expressed genes in *B.mori* hemocytes after the treatment of DA in differential time intervals.

To check whether DA could result in significantly changes of gene expression in *B.mori* hemocytes, we identified and compared the differentially expressed genes between the DA-treated and control samples (Figure S4), which were further calculated by normalizing the number of unambiguous tags in each library to reads per kb per million reads (RPKM). The results revealed that many genes were significantly differentially expressed between the control and treated libraries. Furthermore, all the genes with the expression more than 2-fold changes were annotated by using Nr database, GO database and KEGG pathway database (Table S1). Among them, we found that the toxicity response of *B.mori* to DA was mainly associated with the insect innate immune response, xenobiotic detoxification and apoptosis. Interestingly, the profile of genes number affected by DA showed a curve type with a peak at the treatment of past 12 h (Figure 2).

**Figure 3:**
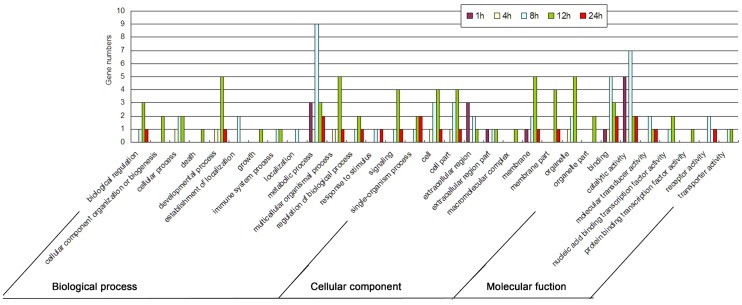
Summarizing of Gene Ontology functional classification of ten DGEs by comparing DA-treated and un-treated samples in differential time intervals, which described gene products in terms of their associated biological processes, cellular components and molecular functions.

At 1 h post-treatment, 10 genes were up-regulated with at least 2-fold changes (Figure 2). Among them, 5 genes of proteases and aminopeptidase (Bm_nscaf2674_066, Bm_nscaf2674_064, Bm_nscaf2674_063, Bm_nscaf2983_049 and Bm_nscaf2889_046) and 2 putative cuticle protein (Bm_nscaf2838_045 and Bm_nscaf2767_133) and the other 3 genes were annotated. It indicated that expression of digest-related genes was mainly changed in hemocytes in their early response to DA treatment. It is in accordance with the Gene Ontology annotation (Figure 3), which showed that genes involving in metabolic process and catalytic activity were much up-regulated at 1 h post treatment. Several signal pathways such as fatty acid metabolism and focal adhesion were also activated (Table S2).

At 4 h post treatments, 20 and 1 genes were up-regulated and down-regulated, respectively (Figure 2). The highest up-regulated gene was Bm_nscaf2767_133 (9.4-fold), which was the only detected up-regulated gene at 4 time points (1 h, 4 h, 8 h and 12 h) and annotated as a putative cuticle protein. Whereas, Bm_nscaf2767_074 gene was the only detected down-regulated gene (-2.0-fold) and annotated as a transposon Ty3-G gap-Pol polyprotein in *Clonorchis sinensis*. Multiple signal transduction pathways were also activated at 4 h post treatment, such as MAPK (Mitogen-activated protein kinases) signaling pathway. Two genes (Bm_nscaf2136_098 and Bm_nscaf2888_274) which participate in MAPK pathway were annotated by KEGG (Table S2).

At 8 h post treatments, there were 18 up-regulated genes and 8 down-regulated genes (Figure 2). Bm_nscaf3058_128 was the highest up-regulated gene with 12.8-fold, which was annotated as peptidoglycan recognition protein (Table S1). Bm_nscaf2589_181 was down-regulated by -2.1-fold and annotated as E3 ubiquitin-protein ligase RNF31. These two genes play a key role in regulating immune and apoptopic signaling [27]. The other genes functions were annotated as well (Figure 2). Several signal pathways such as pancreatic secretion were also activated (Table S2).

At 12 h and 24 h post treatments, 74 and 8 genes were respectively up-regulated, as well as 13 and 3 genes were respectively down-regulated (Figure 2). Bm_nscaf2136_210 and Bm_nscaf2818_080 were the highest induced expression genes at both 12 h and 24 h post treatments, and were annotated as tropomyosin-2 isoform 2 and protease inhibitor 6, respectively. Lots of other genes were mainly found participating in developmental, multicellular organismal and signalling processes (Figure 3). ABC transporters and many other signal pathways were probably activated (Table S2 6).

### Gene expression analysis and qRT-PCR validation

In order to confirm the integrity of the DGE data, qRT-PCR was carried out. Twenty genes were randomly selected including alkaliphilic serine protease, two putative cuticle protein CPH45, tubulointerstitial nephritis antigen precursor, serine protease precursor, Bmp-2 protein, fungal protease inhibitor F precursor and putative serotonin receptor and so on. The PCR products were sequenced, and further confirmed by blasting in NCBI database. The quantification data showed that the eighteen genes expression profile were consistent with the DGE results, although the ratios varied to some extent, but two genes (Bm_nscaf2838_045 and Fungal protease inhibitor F precursor) are not significantly expressed in the checked samples, although both revealed the same expression tendency as DGE results (Table 2).

**Table 2:** Quantitative Real Time PCR (qRT-PCR) validation of DGE result.

Gene ID	Protein	Time after treatment	DGE Log2 ratio	qPCR	
				Primers(5′ to 3′)	Relative expression
Bm_nscaf2674_064	Alkaliphilic serine protease	1 h	9.32	F: GGAGGTTCTGGTTCGGR: GGACATTGTTGTGGAGGA	19.56±2.27^a^
Bm_nscaf2674_063	alkaliphilic serine protease	1 h	5.13	F: ACGACCAACATTAACCAGTA R: TGAGCGGCACTAAGGATA	10.4±1.70^a^
Bm_nscaf2838_045	Putative cuticle protein CPH45	1 h	6.77	F: TGCCATTGTTGAAGGTGC R: GGGTCTCGGGAAGAGTGA	1.21±0.09^b^
Bm_nscaf2818_064	chlorophyllide A binding protein precursor	4 h	5.46	F: TACGCTCTCCTGTATTCTTG R: GGTATATTGGTGTCGCATTG	1.95+0.132^a^
Bm_nscaf2767_133	Putative cuticle protein CPH45	4 h	9.36	F: CGTTGACGAAGCCGAAAT R: TGACAGCGGGTGCGGGAATG	1.50±0.23^a^
Bm_nscaf2795_018	Tubulointerstitial nephritis antigen precursor	8 h	−2.94	F:TAACCAACAAGGGACCAC R: CAATCAGACCCGCACC	−9.04±1.50^a^
Bm_scaffold769_2	Serine protease precursor	8 h	8.90	F:GGCAACCTCCACAAGC R: GGGCACCCAAGTCAGTA	2.78±0.22^a^
Bm_nscaf2983_049	trypsin-like protease precursor	8 h	9.98	F: CGACTACGACAATACTGACT R: AAGAACTGATGCTGGTGAG	7.95±0.84^a^
Bm_nscaf3013_07	putative membrane protein precursor	8 h	10.12	F: CCTCGCAGACAACATCAT R: CGTAGTCCGTCAGGTAGA	3.75±0.45^a^
Bm_nscaf2847_194	threonine 3-dehydrogenase	8 h	−3.67	F: GCGGAGTTGTTAGGAGAG R: TTGCTGATGGCTTCTACC	−2.97±0.14^a^
Bm_nscaf3087_01	hypothetical protein KGM_21246	8 h	−8.69	F: CCAGAATCAACGGCAACA R:TAATGAGGTAGGAAGGAGACT	−5.64±0.66^a^
Bm_nscaf1108_005	hypothetical protein KGM_05290	12 h	7.29	F: GAAGGCGTTGATGGAGAA R: CAAGTCGTAGTCGGAGTG	5.46±0.23^a^
Bm_scaffold721_3	hypothetical protein KGM_18620	12 h	4.26	F: GCGGTAGTCTATGTTGTGA R: TTCGGCTGTAAGGAGTCT	8.27±0.74^a^
Bm_nscaf3003_091	beta-glucosidase precursor	12 h	8.07	F: GTGGAGATGATGCGAGAG R: CGTTGATTAGCCTGTTGTAG	4.52±0.56^a^
Bm_nscaf463_08	beta-N-acetylglucosaminidase 3 precursor	12 h	9.27	F: CGACCTCCTGACACATTG R: AACTTCCTGCCACACTATC	17.3±2.6^a^
Bm_nscaf2818_106	Bmp-2 protein	24 h	4.47	F: CGTCACCGGGCAACAT R: TGACGACGCCGAAACG	2.98±3.7^a^
Bm_nscaf2931_28	Fungal protease inhibitor F precursor	24 h	3.14	F: TGGGTGTCCCGAGAATG R: TGTTACAGTGGCAGGTTGG	1.07±0.11^b^
Bm_nscaf463_19	Putative serotonin receptor	24 h	−2.25	F: GAAGAGCCAAGGACCACT R: ACGGAAGCCAGCAGAA	−1.55±0.13^a^
Bm_nscaf2818_106	bmp-2 protein	24 h	2.54	F: GGCGTTATATCCGTGGAG R: CCGTTAGGTATGTGGTGAG	7.46±0.65
Bm_nscaf3045_59	glucosinolate sulfatase	24 h	−3.07	F: TGACAATGGCGGTATGAC R: TGGACAGAATCAGCATAAGA	−6.43±0.63

a means significant difference existed between the DA treated and control samples.

b means no significant difference.

DGE data were generated from two biological repeats. qPCR data were generated by the standard deviation from three biological repeats.

## Discussion

### Summary of the DGE results

Our DGE data firstly provided a comprehensive and global genes expression profile of *B. mori* hemocytes at different time points after DA treatment. The expression results with at least 2-fold changes showed that the numbers of up-regulated genes are 10, 20, 18, 74 and 8, as well as the numbers of down-regulated genes are 0, 1, 8, 13 and 3 after the treatment of 1 h, 4 h, 8 h, 12 h and 24 h, respectively. To sum up, the expression of 132 genes were significantly changed (in which 1, 2 and 12 genes were continually up-regulated at 4, 3 and 2 different time points, respectively, while 1 gene was either up or down-regulated continually at 2 different time points). Among them, 68 genes were assigned to one or multiple gene ontology (GO) terms and 89 genes were assigned to specific Kyoto Encyclopedia of Genes and Genomes (KEGG) Orthology. To sum up, 108 genes increased expression, while 25 genes decreased. The genes with significantly changed expression in this study were much less than a similarly study in *P. xylostella*, in which 1584 genes were observed with at least 2-fold changes, but comparing both, our data are only from hemocytes of *B. mori*, while the whole insect body of the fourth instar larvae were utilized in the DGE analysis of *P. xylostella*, which should be the reason why much more genes have been detected in *P. xylostella* [28].

### Genes putatively involved in insect immune system

The immune system is generally divided into innate and adaptive immunity. Insect has only innate immunity system divided into humoral and cellular responses [29], [30]. The humoral response of innate immunity mainly includes three steps [31], [32], [33]: 1) identification of pathogen-associated molecular patterns (PAMPs) on pathogens by pattern recognition receptors (PRRs); 2) activation of the regulatory pathways; and 3) production of immune effectors to modulate cellular phagocytosis and molecular effectors such as antimicrobial peptides (AMPs). Peptidoglycan recognition proteins (PGRPs) are pattern recognition molecules that recognize bacteria and their unique cell wall component, peptidoglycan (PGN) [34]. In our study, four PGRPs (Bm_nscaf3058_131, Bm_nscaf3058_127, Bm_nscaf3058_130, Bm_nscaf3058_128) were significantly up-regulated. Specially, Bm_nscaf3058_128 was increased the expression of 12.78-fold after the treatment 8 h, and Bm_nscaf3058_127 was persistently up-regulated past the treatment of 8 h and 12 h with the changes of 2.06-fold and 2.93-fold, respectively (Table S1), indicating that *B. mori* innate immunity response to DA was activated through PGRP at a very early stage. DA also accelerated the expression of PGRPs of larvae of *P. xylostella* [28]. It suggests that PGRPs may be a common PRR that regulate the immunity of insects response to DA.

Insect PGRPs can activate the Toll signal pathway inducing production of antibacterial peptides (AMPs) such as cecropin and gloverin [35], [36]. In our study, *B. mori* Toll receptor was induced the expression of 2.39-fold after DA treatment of 8 h (Table S1). In consistence with our data, Toll was also up-regulated with 2-fold in *P. xylostella* larvae after injecting DA [28]. However, the scavenger receptor and C-type lectin were also up-regulated simultaneously in *P. xylostella* [28], while in our study, the expression changes of scavenger receptor and C-type lectin were not found. That may be caused by activating different immune cell responses to DA in different insect species or tissues, For example, in *M. sexta* hemocytes, Toll mRNA was significantly induced by *Escherichia coli*, *Saccharomyces cerevisiae* and *Micrococcus lysodeikticus*, but its transcript in the fat body was not induced by these microorganisms [37].

There were no significant changes of AMPs in our study, which may be due to either the threshold value of 2-fold is too high to find the difference, or indicating the evolutionary plasticity of *B. mori* by presenting novel proteins correlated with the response to DA [38]. But Pal et al (2007) found that DA could mediate a specific down-regulation of AMPs in *Drosophila melanogaster*, such as cecropins, attacin, metchnikowan, and diptericin [24], which led to a conclusion that DA has the potentiality to suppress the humoral immune response in *D. melanogaster*.

On the other hand, hemocytes are the primary mediators of cell-mediated immunity in insects including phagocytosis, nodulation, encapsulation, and melanization [39]. Phagocytosis is an ancient cellular process that plays an important role in host defense. Bm_nscaf2529_073 is homologue to *D. melanogaster* CG2765 acting as mediators of bacterial phagocytosis in cell line S2 [40]. In our study, this gene was significantly down-regulated. It suggests that gene Bm_nscaf2529_073 has an essential role of phagocytosis during the process of immunity of *B. mori* response to DA.

### Genes putatively involved in insecticide detoxification and outside stress environment

The cytochrome P450 is a large and diverse group of enzymes that catalyze the oxidation of organic substances, such as xenobiotic [41]. Two cytochrome P450 genes, Bm_nscaf3005_56 and Bm_nscaf2827_03, were up-regulated with 4.2-fold and 3.1-fold after the treatment of 8 h, respectively (Table S1). However, in the case of *P. xylostella*, the expression of cytochrome P450 was not found to be changed, while Glutathione S-transferase gene was up-regulated after treated with DA[28].

Insect heat shock proteins (Hsp) are important molecular chaperone which were the first introduced by Tissieres et al. (1974) [42] due to its increased expression to the high temperature in *D. melanogaster*, and it also has been confirmed that Hsp involved in multiple physiological roles such as to increase lifespan, enhance stress resistance, and prevent apoptosis and neurodegenerative diseases [43], [44]. In our study, the expression of one 19.5 kDa Hsp and three 70 kDa Hsps were significantly decreased, but one 20.4 kDa Hsp was significantly increased, which provide a complex physiology role of this family protein in the process of *B. mori* response to DA.

### Genes involved in early and persistently response to DA

In our experiment, numerous data of gene expression at five different time points (1 h, 4 h, 8 h, 12 and 24 h) after DA treatment were accomplished. However, we think that the genes involved in the early and continually response to DA should be paid more attention. *B. mori* cuticle proteins play essential roles in many physiological functions, during molting and metamorphosis [45]. Interestingly, two cuticle protein genes were found continual up-regulation in this research. Bm_nscaf2767_133 gene continually increased the expression with 4.62-fold, 9.36-fold, 9.69-fold and 4.7-fold in post-treatment 1, 4, 8 and 12 h, respectively, while Bm_nscaf2838_045 gene were up-regulated 6.8-fold in 1 h, 11.0-fold in 8 h and 4.7-fold in 12 h (Table S1). In consistence with our data, Zhang et al. (2010) [46] reported that the putative cuticle protein genes in adult of *Leptinotarsa decemlineata* could be highly induced by either insecticide azinphosmethyl (a type of organophosphate insecticide) 2–3 weeks after moulting, or dry environmental conditions. Consequently, we believe that insect cuticle proteins perhaps have other unknown functions.

Four trypsin genes (Bm_nscaf2674_064, Bm_nscaf2674_063, Bm_nscaf2983_049 and Bm_nscaf2674_066) were all significantly up-regulated at early or persistent time points. Trypin is a common serine protease involved in protein precursor cleavage used for signal transduction and cascade amplification, and can also activate specific defense mechanisms, such as complement activation, melanization, blood coagulation and antibacterial peptide synthesis [47]. So, we can hypothesize that DA stimulates silkworm hemocytes expressing immediately trypsins to start some signal transductions.

### In conclusion

The most important finding in this study gives an overview of genes expression profile of *B. mori* hemocytes after the treatment of DA during different intervals, which can help to better understanding the immune response of *B. mori* hemocytes underling the stress of DA. But the results also highlight the physiological role of some proteins of *B. mori* response to DA, such as heat shock protein and cuticle protein. Taken them together, our findings are helpful to elucidate the molecular mechanisms of DA as a biologic insecticide.

## Methods

### Insects

The strain of *Bombyx mori*, P50, was provided by Dr. Ye Mingqiang (Sericulture&Agri-food Research Institute, Guangdong Academy Agricultural Science). The one-day old fifth instar larvae of silkworms were collected and used in this study.

### Destruxin A and chemicals

Destruxins A (DA) was isolated and purified from strain MaQ10 of *Metarhizium anisopliae* as described [48], [49]. DA was identified and determined with purification rate of 95.7% by means of high performance liquid chromatography (HPLC) with a standard sample from Sigma-Aldrich Co. LLC (St. Louis, MO, USA) [23]. DA was diluted to 10000 µg/mL using dimethyl sulfoxide (DMSO, Sigma-Aldrich Co. LLC) as the stock solutions and stored at −20°C for further use.

### Injection of DA into Silkworm Larvae

Firstly, DA stock was diluted with double distilled water to be 200 µg/mL. The injection dose of DA to each 5th instar larva was 10 µL. Before injection, the larvae of *Bombyx mori* were placed on ice for 5 min to paralyze. Before injection, the insect's surface were carefully washed with pure water, then, disinfected with a small amount of alcohol. Then, the needle tip was inserted in the soft part of prolegs of paralytic silkworm larvae and 10 µL DA working solution was carefully injected into silkworm larvae. Then, the injected larvae were maintained at 26°C. The control group was treated with 1% DMSO aqueous solution.

### Collection of hemocytes

Hemocytes were collected 1, 4, 8, 12 and 24 h after injection, samples at each time point were dissected from 5 fifth instar larvae of *B. mori*. Before bleeding, the insect's surface were carefully washed with pure water, then, disinfected with a small amount of alcohol. When bleeding, the insect was placed on ice for a few minutes to paralyze, then, its hind leg was cut with a surgical scissors, and the blood was dripped into a centrifuge tube containing 650 µL anticoagulation buffer (KCl 69 mmol/L, NaCl 27 mmol/L, NaHCO_3_ 2 mmol/L, dextrose 100 mmol/L, potassium citrate 30 mmol/L, citrate acid 26 mmol/L, Na_2_-EDTA 10 mmol/L respectively, and pH 4.6, 420 mosm.) on ice. Then, the mixture of blood and buffer was centrifuged at the speed of 1000 r/min for 10 min at 4°C and the supernatant was abandoned. One ml RNAiso Plus (Trizol, Takara biotechnology (Dalian) CO., LTD) was added to the hemocytes and stored at −80°C for further use.

### Gene expression profile sequencing

All collected hemocyte samples were used for RNA isolation. Total RNA from these samples was extracted using Trizol Total RNA Isolation Kit (Takara, Japan) according to manufacturer's protocol, followed by quality and quantity analysis using Nanodrop (Bio-Rad, USA) and 2100 Bioanalyzer (Agilent, USA). Digital gene expression (DGE) libraries were prepared by the Illumina gene expression sample prep kit (Illumina, San Diego, CA). Six micrograms of total RNA was used to isolate mRNAwith Oligo(dT) magnetic beads adsorption. The first and second cDNAs were synthesized and the bead-bound cDNAs were subsequently digested by Nla III, which recognizes and cuts on the CATG sites. The digested 3′cDNA fragments were purified with magnetic Beads, and connected to the Illumina adapter 1 at the sticky 5′-ends. The junction of adapter 1 and CATG site is the recognition site of Mme I, which cuts off the cDNA at a position of 17 bp downstream of the CATG site, thereby producing tags with adapter 1. After removing 3′-end fragments with magnetic beads precipitation, Illumina adapter 2 was ligated to the tags at the 3′-ends, generating tags with different adapters at both ends to form a tag library. The fragments are enriched by PCR amplification, 95 bp bands were purified by 6% PAGE gel electrophoresis. After denaturation, the single-stranded molecules were fixed onto the Illumina sequencing chip (flow cell) for sequencing by using the sequencing by synthesis (SBS) method. Each tunnel generated millions of raw 49 bp reads. Each time point provides two samples for sequencing as two biological replicates. The data sets are deposited at the NCBI Short Read Archive (http://www.ncbi.nlm.nih.gov/sra/) with an accession number for the treatment at each time point (Table 3).

**Table 3:** NCBI SRA accession numbers for the treatments at each time point.

Name	Past the treatment	Accession number
1hCK	1 h	SRX480635
1hDA	1 h	SRX480636
4hCK	4 h	SRX480637
4hDA	4 h	SRX480638
8hCK	8 h	SRX480639
8hDA	8 h	SRX480640
12hCK	12 h	SRX480641
12hDA	12 h	SRX480642
24hCK	24 h	SRX480643
24hDA	24 h	SRX480644

### Bioinformatics analysis of digital gene expression (DGE) tags

Before mapping the tags to the transcriptome database, the raw sequence data were filtered by removing low quality tags, such as tags with unknown sequences ‘N’, empty tags (reads with only adapter sequences but no tags), low complexity tags, and tags with a copy number of 1 (likely sequencing errors). A reference library containing all of the sequences of CATG plus 17 bases was created by searching the CATG sites in the transcriptome database. All clean tags were mapped to the reference library and allowed no more than one base mismatch. Clean tags, which were mapped to exactly one gene in the reference database, were designated as unambiguous tags for gene annotation. The number of unambiguous tags for each gene was calculated and normalized to RPKM (Reads Per Kb per Million reads) for the gene expression analysis [50], [51].

### Screening and statistics analysis of differentially expressed genes

A rigorous algorithm was developed to identify differentially expressed genes (DEGs) in both treatment and control conditions. The total clean tag number of the sample 1 is N1, and total clean tag number of sample 2 is N2; gene A holds x tags in sample1 and y tags in sample2. The probability of gene expressed equally between two samples can be calculated with:
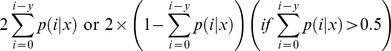





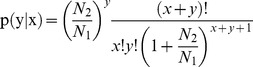
The false discovery rate (FDR) method was used to determine the P-value threshold in multiple testing [52]. FDR≤0.001 and the absolute value of log2^ratio^ ≥2 were used as the threshold to judge the significance of gene expression differences. For Gene Ontology (GO) enrichment analysis, we used the hypergeometric test to map all the differentially expressed genes to terms in the GO database, identifying GO terms significantly enriched for DEGs, and comparing them to the genome background.
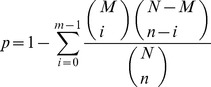
where N is the number of all genes with GO annotation; n is the number of DEGs in N; M is the number of all genes that are annotated to the certain GO terms; m is the number of DEGs in M. The calculated p-value goes through Bonferroni Correction, taking corrected p-value ≤0.05 as a threshold. GO terms fulfilling this condition are defined as significantly enriched GO terms in DEGs. This analysis is able to recognize the main biological functions that DEGs exercise.

The differentially expressed genes were also utilized in Kyoto Encyclopedia of Genes and Genomes (KEGG) ontology enrichment analyses to further understand their biological functions. KEGG Pathway analysis can identify significantly enriched metabolic pathways or signal transduction pathways in DEGs compared with the genome background. Pathways with Q-value≤0.05 were considered as significantly enriched in DEGs.

### Validation of gene expression by quantitative real-time PCR

Quantitative real-time reverse transcription PCR (qRT-PCR) was performed to validate the Illumina sequencing data. Eight genes were randomly chosen for qRT-PCR analysis, which have the significantly differential expression in DGE data. The primers are shown in Table 2. Total RNA sample was extracted for the DGE experiment with three biological replicates. The RT-PCR was performed using the AMV RNA Kit (TaKaRa, Japan) according to the manufacturer's protocol. qRT-PCR reactions were performed in triplicate on a BioRad iQ5 real-time PCR detection system using 10 ng of cDNA, 0.2 µM of primers and SYBR Premix Ex TaqTM (TaKaRa) according to the manufacturer's protocol. *Bombyx mori* β-actin was used as a reference gene to standardize the level of other transcripts. The relative amounts of the transcripts were first normalized to the endogenous reference gene and then normalized to the gene expression level in the un-treated samples according to the 2^−ΔΔ^Ct method [53], statistical analysis was performed using Sigma Plot 12.0 software based on t-test with p<0.05 representing significance.

## Supporting Information

Figure S1 **Sequence saturation analysis of each library.**
(TIF)

Figure S2 **Randomness assessment of each library.**
(TIF)

Figure S3 **Distribution of genes' coverage in each library.**
(TIF)

Figure S4 **Scattered plot of differential expression genes in DA-treated and un-treated samples in differential time intervals.** RPKM means Reads Per Kb per Million read.
(TIF)

Table S1 **Genes with more than 2-fold expression changes between the DA-treated and control samples are annotated by using Nr database, GO database and KEGG pathway.**
(DOC)

Table S2 **Details of KEGG pathway enrichment analysis of the genes with significant expression change.**
(DOC)
