# Supplementary figures and images for: Gene Expression Profile of Bombyx mori Hemocyte under the Stress of Destruxin A
Source: PLoS One. 2014 May 6;9(5):e96170. doi: 10.1371/journal.pone.0096170 (PMC4011735; doi:10.1371/journal.pone.0096170)

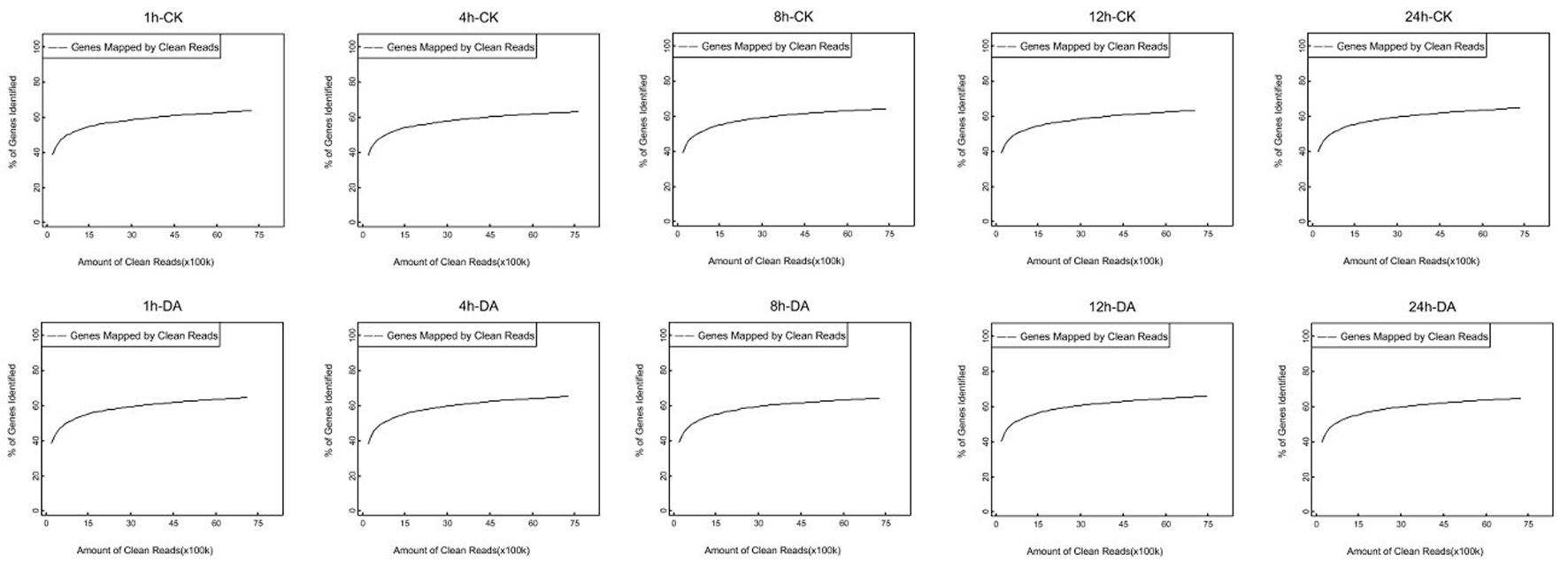

Supplement: Figure S1 — Sequence saturation analysis of each library. (TIF) [file pone.0096170.s001.tif]

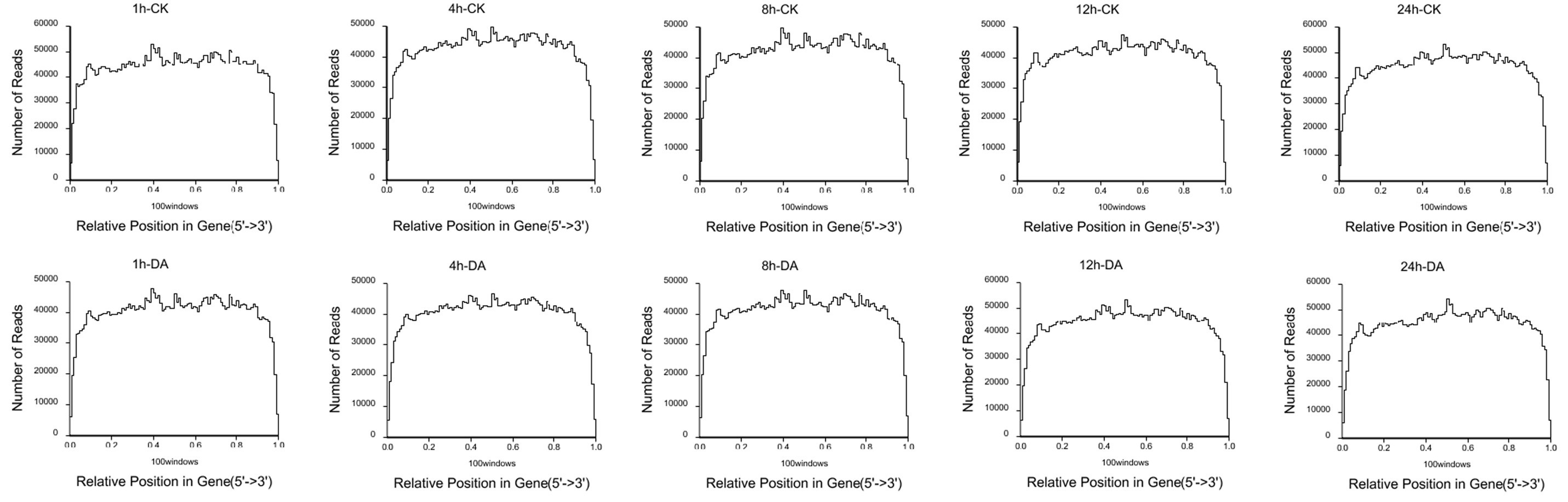

Supplement: Figure S2 — Randomness assessment of each library. (TIF) [file pone.0096170.s002.tif]

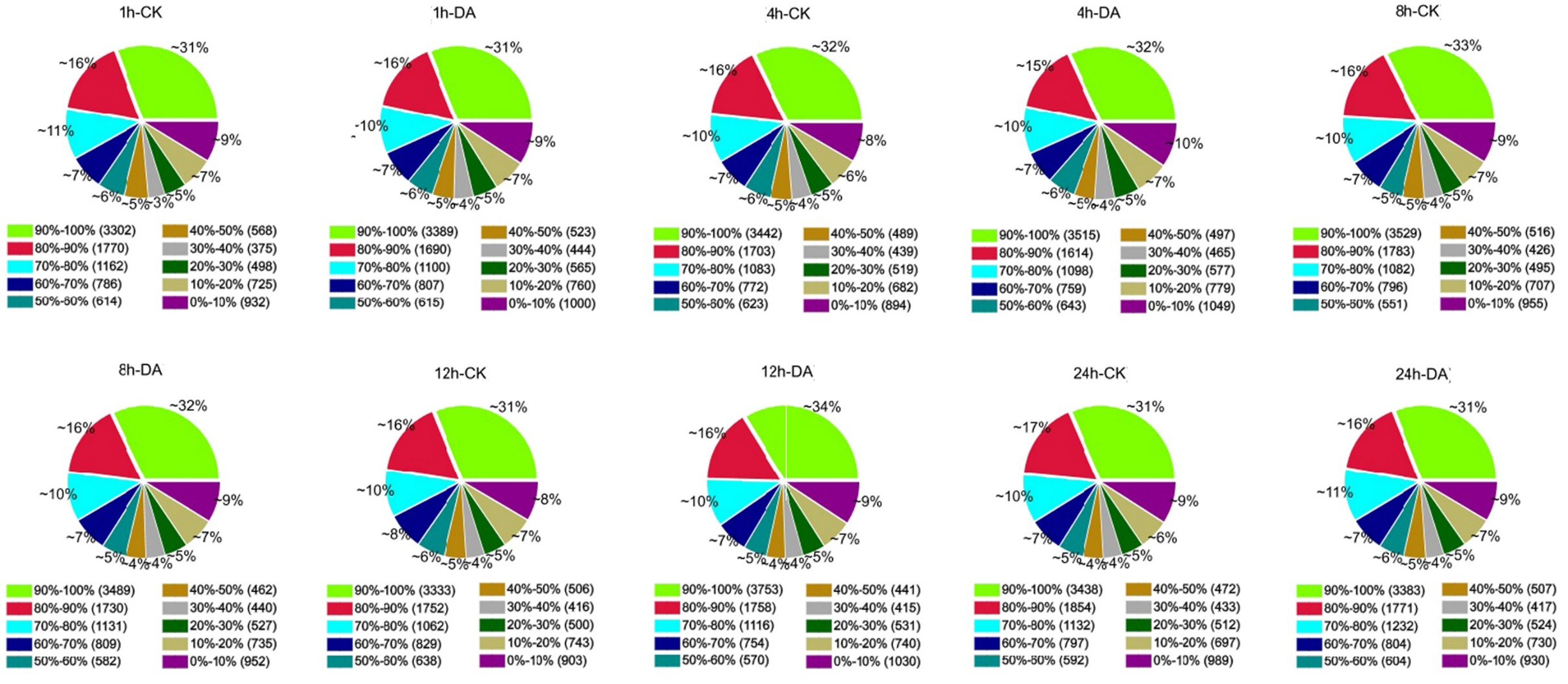

Supplement: Figure S3 — Distribution of genes' coverage in each library. (TIF) [file pone.0096170.s003.tif]

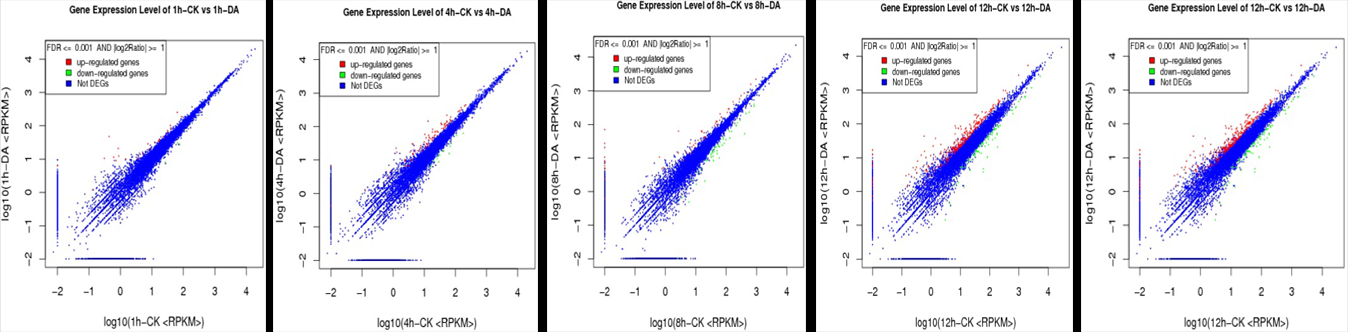

Supplement: Figure S4 — Scattered plot of differential expression genes in DA-treated and un-treated samples in differential time intervals. RPKM means Reads Per Kb per Million read. (TIF) [file pone.0096170.s004.tif]
