# Supplementary material for: Gene Expression Profile of Bombyx mori Hemocyte under the Stress of Destruxin A
Source: PLoS One. 2014 May 6;9(5):e96170. doi: 10.1371/journal.pone.0096170 (PMC4011735; doi:10.1371/journal.pone.0096170)
Supplement: Table S1 — Genes with more than 2-fold expression changes between the DA-treated and control samples are annotated by using Nr database, GO database and KEGG pathway. (DOC) [file pone.0096170.s005.doc]

Supplementary file 5 Genes with the expression more than 2-fold changes and annotated by using Nr database, GO database and KEGG pathway database

| NO. | GeneID | Gene  length | Up/down-regulation | | | | | KEGG Orthology | GO | | | Blast nr |
| --- | --- | --- | --- | --- | --- | --- | --- | --- | --- | --- | --- | --- |
|  |  |  | 1h | 4h | 8h | 12h | 24h |  | Component | Function | Process |  |
| 1 | Bm_nscaf2674_064 | 771 | 9.3 |  |  |  |  | K01312|1|6e-39|161|tca:655678|trypsin [EC:3.4.21.4] | GO:0005576//extracellular region | GO:0070011 | - | gi|402483747|gb|AFQ59994.1|/3.15357e-133/alkaliphilic serine protease [Bombyx mori] |
| 2 | Bm_nscaf2838_045 | 546 | 6.8 |  | 11.0 | 4.7 |  | - | - | - | - | gi|398359539|gb|AFO83995.1|/3.08271e-30/putative cuticle protein CPH45 [Antheraea yamamai] |
| 3 | Bm_nscaf2674_063 | 771 | 5.1 |  |  |  |  | K01312|1|7e-42|171|ame:413645|trypsin [EC:3.4.21.4] | GO:0044421//extracellular region part | GO:0004175//endopeptidase activity | GO:0019538//protein metabolic process | gi|402483747|gb|AFQ59994.1|/1.51592e-127/alkaliphilic serine protease [Bombyx mori] |
| 4 | Bm_nscaf2767_133 | 558 | 4.6 | 9.4 | 9.7 | 4.7 |  | - | - | - | - | gi|398359539|gb|AFO83995.1|/7.95139e-29/putative cuticle protein CPH45 [Antheraea yamamai] |
| 5 | Bm_nscaf2983_049 | 774 | 4.2 |  | 9.0 | 3.9 |  | K01312|1|6e-19|94.7|dmo:Dmoj_GI21247|trypsin [EC:3.4.21.4] | - | GO:0004175//endopeptidase activity | GO:0019538//protein metabolic process | gi|153792257|ref|NP_001093273.1|/3.89411e-123/trypsin-like protease precursor [Bombyx mori] |
| 6 | Bm_nscaf2674_066 | 804 | 3.8 |  | 8.9 |  |  | K01312|1|3e-35|113|ame:413645|trypsin [EC:3.4.21.4] | GO:0005576//extracellular region | GO:0070011 | - | gi|402483747|gb|AFQ59994.1|/2.40086e-133/alkaliphilic serine protease [Bombyx mori] |
| 7 | Bm_nscaf3013_07 | 657 | 3.7 |  | 10.0 |  |  | - | - | - | - | gi|375151601|ref|NP_001243511.1|/1.28492e-43/putative membrane protein precursor [Bombyx mori] |
| 8 | Bm_nscaf2889_046 | 5487 | 3.2 |  |  |  |  | K11140|1|1e-144|516|phu:Phum_PHUM412200|aminopeptidase N [EC:3.4.11.2] | GO:0016020//membrane | GO:0046914//transition metal ion binding;GO:0008238//exopeptidase activity | GO:0019538//protein metabolic process | gi|112983238|ref|NP_001037013.1|/0/aminopeptidase N precursor [Bombyx mori] |
| 9 | Bm_nscaf2847_345 | 642 | 2.8 |  |  |  |  | - | - | - | - | gi|389608415|dbj|BAM17817.1|/5.74255e-33/unknown secreted protein [Papilio xuthus] |
| 10 | Bm_nscaf2931_45 | 1224 | 2.0 |  |  |  |  | - | - | - | - | gi|357625492|gb|EHJ75917.1|/1.13108e-132/hypothetical protein KGM_06296 [Danaus plexippus] |
| 11 | Bm_nscaf2818_064 | 8418 |  | 5.4 |  | 6.2 |  | - | - | - | - | gi|112983040|ref|NP_001037071.1|/0/chlorophyllide A binding protein precursor [Bombyx mori] |
| 12 | Bm_nscaf2847_280 | 198 |  | 4.2 |  |  |  | - | - | - | - | - |
| 13 | Bm_nscaf3097_54 | 753 |  | 3.7 |  |  |  | K01362|1|5e-42|171|dpo:Dpse_GA18526|[EC:3.4.21.-];K09640|3|3e-33|142|api:100166365|transmembrane protease, serine 9 [EC:3.4.21.-] | - | GO:0003824//catalytic activity | - | gi|56418401|gb|AAV91008.1|/4.86599e-83/hemolymph proteinase 10 [Manduca sexta] |
| 14 | Bm_nscaf2953_010 | 738 |  | 3.4 |  | 3.7 | 2.0 | - | - | - | - | - |
| 15 | Bm_nscaf1108_005 | 3990 |  | 3.4 |  | 7.2 |  | K15187|1|2e-07|59.7|dmo:Dmoj_GI22931|YEATS domain-containing protein 1/3 | - | - | - | gi|357627434|gb|EHJ77122.1|/0/hypothetical protein KGM_05290 [Danaus plexippus] |
| 16 | Bm_nscaf2852_094 | 1482 |  | 2.9 |  | 3.4 |  | - | - | - | - | gi|389612129|dbj|BAM19586.1|/0/similar to CG13607, partial [Papilio xuthus] |
| 17 | Bm_nscaf2136_098 | 3024 |  | 2.8 |  |  |  | K07365|1|5e-10|67.8|hmg:100201881|NCK adaptor protein;K04364|2|2e-08|62.8|phu:Phum_PHUM423150|growth factor receptor-binding protein 2 | - | - | - | gi|357625307|gb|EHJ75795.1|/0/hypothetical protein KGM_07342 [Danaus plexippus] |
| 18 | Bm_nscaf2655_149 | 2010 |  | 2.5 |  | 2.7 |  | K08110|1|5e-66|253|dpo:Dpse_GA16712|glypican 4 (K-glypican);K08112|2|3e-55|217|bfo:BRAFLDRAFT_59323|glypican 6 | GO:0016020//membrane | - | - | gi|357627225|gb|EHJ76981.1|/0/glypican [Danaus plexippus] |
| 19 | Bm_nscaf2847_138 | 372 |  | 2.5 |  |  |  | - | - | - | - | gi|357621460|gb|EHJ73283.1|/8.56208e-44/hypothetical protein KGM_07238 [Danaus plexippus] |
| 20 | Bm_scaffold721_3 | 3045 |  | 2.3 |  | 4.2 |  | - | - | - | - | gi|357622602|gb|EHJ74028.1|/0/hypothetical protein KGM_18620 [Danaus plexippus] |
| 21 | Bm_nscaf2800_73 | 546 |  | 2.2 |  |  |  | K09542|1|1e-39|162|aag:AaeL_AAEL013341|crystallin, alpha B | GO:0030529//ribonucleoprotein complex | - | GO:0006950//response to stress | gi|112983152|ref|NP_001037038.1|/4.52183e-90/heat shock protein 20.4 [Bombyx mori] |
| 22 | Bm_nscaf3031_265 | 921 |  | 2.1 |  | 2.2 |  | K06240|1|3e-14|79.7|tca:661583|laminin, alpha 3/5 | - | - | - | gi|357628637|gb|EHJ77904.1|/1.91582e-48/hypothetical protein KGM_21006 [Danaus plexippus] |
| 23 | Bm_nscaf2888_193 | 5085 |  | 2.1 |  |  |  | K06236|1|7e-89|330|phu:Phum_PHUM430790|collagen, type I/II/III/V/XI, alpha | - | - | - | gi|357625680|gb|EHJ76043.1|/0/putative Collagen alpha-1(XI) chain precursor [Danaus plexippus] |
| 24 | Bm_nscaf2888_274 | 3429 |  | 2.1 |  |  |  | K05315|1|0.0|750|cel:T02C5.5|voltage-dependent calcium channel alpha 1, invertebrate | GO:0044456//synapse part;GO:0044459//plasma membrane part | GO:0005262//calcium channel activity | GO:0007626//locomotory behavior;GO:0008016//regulation of heart contraction;GO:0008049//male courtship behavior;GO:0009584//detection of visible light;GO:0016192//vesicle-mediated transport;GO:0070838//divalent metal ion transport;GO:0050804//regulation o | gi|357615821|gb|EHJ69848.1|/0/voltage-dependent p/q type calcium channel [Danaus plexippus] |
| 25 | Bm_nscaf3099_002 | 1131 |  | 2.1 |  |  |  | K05692|1|0.0|763|cqu:CpipJ_CPIJ016462|actin beta/gamma 1 | GO:0043232 | GO:0032559 | - | gi|157122933|ref|XP_001659963.1|/0/actin [Aedes aegypti] |
| 26 | Bm_nscaf2216_03 | 954 |  | 2.0 |  | 2.0 |  | - | GO:0043229//intracellular organelle | GO:0005515//protein binding;GO:0004386//helicase activity | GO:0009987//cellular process;GO:0043170;GO:0032501//multicellular organismal process;GO:0032502//developmental process | gi|262034597|gb|ACY06923.1|/1.45173e-147/putative cuticle protein CPG44 [Bombyx mori] |
| 27 | Bm_nscaf2589_196 | 2244 |  | 2.0 |  | 2.3 |  | K05625|1|9e-115|415|mdo:100032853|transglutaminase 2 [EC:2.3.2.13];K05619|2|5e-114|412|spu:578380|transglutaminase 1 [EC:2.3.2.13] | - | GO:0016740//transferase activity | GO:0006959//humoral immune response | gi|357611653|gb|EHJ67590.1|/0/Annulin [Danaus plexippus] |
| 28 | Bm_nscaf2998_07 | 1029 |  | 2.0 |  |  |  | K00757|1|2e-135|482|phu:Phum_PHUM411930|uridine phosphorylase [EC:2.4.2.3] | GO:0044424//intracellular part | GO:0016763//transferase activity, transferring pentosyl groups | GO:0009117//nucleotide metabolic process | gi|357627849|gb|EHJ77397.1|/2.30333e-141/hypothetical protein KGM_04686 [Danaus plexippus] |
| 29 | Bm_nscaf2511_135 | 657 |  | 2.0 |  |  |  | - | - | - | - | gi|357619405|gb|EHJ71995.1|/8.919e-37/hypothetical protein KGM_00654 [Danaus plexippus] |
| 30 | Bm_nscaf2767_074 | 366 |  | -2.0 |  |  |  | - | - | - | - | gi|358341466|dbj|GAA49140.1|/9.92647e-16/transposon Ty3-G gap-Pol polyprotein [Clonorchis sinensis] |
| 31 | Bm_nscaf3058_128 | 207 |  |  | 12.8 |  |  | K01446|1|6e-15|79.3|tca:658050|N-acetylmuramoyl-L-alanine amidase [EC:3.5.1.28] | - | - | - | gi|379698954|ref|NP_001243949.1|/1.21803e-29/peptidoglycan recognition protein precursor [Bombyx mori] |
| 32 | Bm_nscaf2943_017 | 591 |  |  | 9.5 |  |  | - | - | - | - | gi|13383195|dbj|BAB39500.1|/2.51752e-10/fibroin P25 [Bombyx mandarina] |
| 33 | Bm_nscaf2529_052 | 999 |  |  | 9.2 |  |  | K01046|1|9e-33|141|dme:Dmel_CG17191|triacylglycerol lipase [EC:3.1.1.3] K01058 phospholipase A1 [EC:3.1.1.32];K14075|2|1e-30|134|aml:100471180|pancreatic lipase-related protein 2 [EC:3.1.1.3];K13618|3|5e-30|132|mgp:100546329|phospholipase A1 member A [EC:3.1.1.-] | - | GO:0003824//catalytic activity | GO:0044238//primary metabolic process | gi|112983352|ref|NP_001036966.1|/1.7825e-167/lipase-1 [Bombyx mori] |
| 34 | Bm_scaffold769_2 | 855 |  |  | 8.9 |  |  | K01310|1|3e-36|152|dpo:Dpse_GA19618|chymotrypsin [EC:3.4.21.1];K09640|2|4e-29|129|dse:Dsec_GM14740|transmembrane protease, serine 9 [EC:3.4.21.-] | - | GO:0004175//endopeptidase activity | GO:0019538//protein metabolic process | gi|112984052|ref|NP_001036826.1|/1.54491e-134/serine protease precursor [Bombyx mori] |
| 35 | Bm_nscaf2930_184 | 2112 |  |  | 7.9 |  |  | K00505|1|1e-52|208|der:Dere_GG22822|tyrosinase [EC:1.14.18.1] | GO:0044421//extracellular region part | GO:0022892 | GO:0015669//gas transport | gi|124430725|ref|NP_001037590.1|/0/sex-specific storage-protein 2 precursor [Bombyx mori] |
| 36 | Bm_scaffold792_1 | 8607 |  |  | 6.0 |  |  | K14616|1|0.0|1161|ame:411053|cubilin | GO:0043231//intracellular membrane-bounded organelle;GO:0044444//cytoplasmic part;GO:0016020//membrane | GO:0005488//binding | GO:0006810//transport | gi|357612776|gb|EHJ68165.1|/0/cubilin [Danaus plexippus] |
| 37 | Bm_nscaf3058_131 | 186 |  |  | 4.5 |  |  | K01446|1|8e-07|52.4|dan:Dana_GF10680|N-acetylmuramoyl-L-alanine amidase [EC:3.5.1.28] | - | - | - | gi|379698954|ref|NP_001243949.1|/3.86766e-12/peptidoglycan recognition protein precursor [Bombyx mori] |
| 38 | Bm_nscaf2767_111 | 789 |  |  | 4.1 |  |  | - | - | - | - | gi|294939821|ref|XP_002782573.1|/4.32411e-08/conserved hypothetical protein [Perkinsus marinus ATCC 50983] |
| 39 | Bm_nscaf2855_239 | 2889 |  |  | 2.6 |  |  | K12047|1|2e-41|171|xtr:100379714|maltase-glucoamylase [EC:3.2.1.20 3.2.1.3];K05011|2|6e-10|67.4|dre:100150892|chloride channel 2;K10901|3|3e-09|65.1|api:100165064|bloom syndrome protein [EC:3.6.4.12] | - | - | - | gi|328716627|ref|XP_003245995.1|/8.94237e-138/PREDICTED: hypothetical protein LOC100570266 [Acyrthosiphon pisum] |
| 40 | Bm_nscaf3035_065 | 1440 |  |  | 2.6 |  |  | K14074|1|5e-33|142|acs:100555866|pancreatic lipase-related protein 1 [EC:3.1.1.3];K14073|2|5e-33|142|tca:657679|pancreatic triacylglycerol lipase [EC:3.1.1.3];K14075|5|7e-32|139|bta:510772|pancreatic lipase-related protein 2 [EC:3.1.1.3] | - | - | - | gi|270001417|gb|EEZ97864.1|/3.31099e-65/hypothetical protein TcasGA2_TC000236 [Tribolium castaneum] |
| 41 | Bm_scaffold416_04 | 1125 |  |  | 2.4 |  |  | K06260|1|8e-07|55.5|ecb:100060025|platelet glycoprotein V | - | - | - | gi|126635756|gb|ABO21763.1|/2.82041e-18/toll receptor [Manduca sexta] |
| 42 | Bm_nscaf3058_127 | 387 |  |  | 2.1 | 2.9 |  | K01446|1|4e-33|139|tca:658050|N-acetylmuramoyl-L-alanine amidase [EC:3.5.1.28] | - | GO:0016811//hydrolase activity, acting on carbon-nitrogen (but not peptide) bonds, in linear amides | GO:0000270//peptidoglycan metabolic process | gi|112983866|ref|NP_001036858.1|/1.47394e-72/peptidoglycan recognition protein S6 precursor [Bombyx mori] |
| 43 | Bm_nscaf3058_130 | 210 |  |  | 2.1 |  |  | K01446|1|5e-15|79.7|tca:658050|N-acetylmuramoyl-L-alanine amidase [EC:3.5.1.28] | - | - | - | gi|379698954|ref|NP_001243949.1|/6.4411e-31/peptidoglycan recognition protein precursor [Bombyx mori] |
| 44 | Bm_nscaf2589_181 | 6054 |  |  | -2.1 |  |  | K11974|1|9e-41|171|tca:663619|E3 ubiquitin-protein ligase RNF31 [EC:6.3.2.19] | - | - | - | gi|357622896|gb|EHJ74256.1|/0/hypothetical protein KGM_01628 [Danaus plexippus] |
| 45 | Bm_nscaf2930_138 | 2319 |  |  | -2.5 |  |  | K11423|1|5e-08|60.8|dgr:Dgri_GH16034|histone-lysine N-methyltransferase SETD2 [EC:2.1.1.43];K10352|2|4e-07|57.8|cfa:479497|myosin heavy chain;K10357|3|9e-07|56.6|mmu:208943|myosin V | - | - | - | gi|357609606|gb|EHJ66539.1|/0/hypothetical protein KGM_18481 [Danaus plexippus] |
| 46 | Bm_nscaf1690_227 | 510 |  |  | -2.6 |  |  | K08705|1|1e-42|172|nvi:100115727|nuclear receptor subfamily 5 group A member 3 | GO:0043231//intracellular membrane-bounded organelle | GO:0046914//transition metal ion binding;GO:0001071//nucleic acid binding transcription factor activity;GO:0003677//DNA binding;GO:0004879//ligand-activated sequence-specific DNA binding RNA polymerase II transcription factor activity | GO:0009755//hormone-mediated signaling pathway;GO:0006351//transcription, DNA-dependent | gi|341926128|dbj|BAK53999.1|/1.15021e-83/nuclear hormone receptor [Bombyx mori] |
| 47 | Bm_nscaf2589_120 | 927 |  |  | -2.7 |  |  | K10693|1|3e-08|59.7|dpe:Dper_GL26848|E3 ubiquitin-protein ligase MYCBP2 [EC:6.3.2.19];K01104|2|3e-07|56.2|tad:TRIADDRAFT_54170|protein-tyrosine phosphatase [EC:3.1.3.48] | - | - | - | gi|357626207|gb|EHJ76377.1|/6.076e-135/putative CRAL/TRIO domain-containing protein [Danaus plexippus] |
| 48 | Bm_nscaf2795_018 | 717 |  |  | -2.9 |  |  | K01363|1|3e-30|132|bfo:BRAFLDRAFT_247264|cathepsin B [EC:3.4.22.1];K01275|4|6e-26|117|bfo:BRAFLDRAFT_125840|cathepsin C [EC:3.4.14.1] | - | GO:0001871;GO:0004175//endopeptidase activity;GO:0004888//transmembrane signaling receptor activity | GO:0019538//protein metabolic process;GO:0002376//immune system process | gi|182509202|ref|NP_001116812.1|/3.34705e-139/tubulointerstitial nephritis antigen precursor [Bombyx mori] |
| 49 | Bm_nscaf2823_071 | 372 |  |  | -3.3 |  |  | - | - | - | - | gi|357605803|gb|EHJ64784.1|/7.44874e-64/hypothetical protein KGM_12063 [Danaus plexippus] |
| 50 | Bm_nscaf2847_194 | 1038 |  |  | -3.6 |  |  | K15789|1|6e-141|500|dan:Dana_GF10346|threonine 3-dehydrogenase [EC:1.1.1.103] | - | GO:0048037//cofactor binding | GO:0008152//metabolic process | gi|112982820|ref|NP_001037542.1|/1.31426e-168/L-threonine dehydrogenase [Bombyx mori] |
| 51 | Bm_nscaf3087_01 | 1593 |  |  | -8.0 |  | 3.7 | K08144|1|5e-37|156|mdo:100015097|MFS transporter, SP family, solute carrier family 2 (facilitated glucose transporter), member 6;K14258|5|2e-34|147|isc:IscW_ISCW024553|facilitated trehalose transporter | - | - | - | gi|357623511|gb|EHJ74627.1|/2.03048e-135/hypothetical protein KGM_21246 [Danaus plexippus] |
| 52 | Bm_nscaf2767_130 | 447 |  |  |  | 6.2 |  | - | - | - | - | gi|262034621|gb|ACY06935.1|/8.46143e-36/putative cuticle protein CPH40 [Bombyx mori] |
| 53 | Bm_nscaf2589_115 | 648 |  |  |  | 2.0 |  | K01104|1|4e-09|61.6|tgu:100219399|protein-tyrosine phosphatase [EC:3.1.3.48] | GO:0044464//cell part | - | GO:0051234//establishment of localization | gi|357623555|gb|EHJ74659.1|/1.08473e-103/putative CRALBP [Danaus plexippus] |
| 54 | Bm_nscaf2827_03 | 819 |  |  |  | 3.1 |  | K15004|1|7e-08|58.2|nvi:100121218|cytochrome P450, family 12 [EC:1.14.-.-] | - | - | - | gi|357616868|gb|EHJ70454.1|/4.68175e-61/cytochrome P450 333B11 [Danaus plexippus] |
| 55 | Bm_nscaf3072_46 | 1164 |  |  |  | 2.1 |  | K12114|1|8e-79|294|nvi:100116208|vrille | - | GO:0005488//binding | - | gi|46403173|gb|AAS92609.1|/1.11506e-150/vrille [Antheraea pernyi] |
| 56 | Bm_nscaf2589_114 | 234 |  |  |  | 2.1 |  | - | - | - | - | gi|357623554|gb|EHJ74658.1|/2.32473e-17/putative CRALBP [Danaus plexippus] |
| 57 | Bm_nscaf2204_083 | 1152 |  |  |  | 2.1 |  | K00540|1|5e-65|248|hsa:10493|[EC:1.-.-.-];K10133|3|6e-23|108|xla:444383|tumor protein p53-inducible protein 3 [EC:1.-.-.-] | - | GO:0016655//oxidoreductase activity, acting on NADH or NADPH, quinone or similar compound as acceptor;GO:0046914//transition metal ion binding | GO:0008152//metabolic process | gi|153792203|ref|NP_001093281.1|/2.20116e-167/vesicle amine transport protein [Bombyx mori] |
| 58 | Bm_nscaf2511_133 | 795 |  |  |  | 2.5 |  | - | - | - | - | gi|167736344|ref|NP_001108066.1|/7.17982e-152/spatzle-1 precursor [Bombyx mori] |
| 59 | Bm_nscaf2818_080 | 261 |  |  |  |  | 10.8 | - | - | - | - | gi|56462340|gb|AAV91453.1|/6.18155e-26/protease inhibitor 6 [Lonomia obliqua] |
| 60 | Bm_nscaf2136_210 | 267 |  |  |  | 10.7 |  | K10373|1|6e-34|142|aag:AaeL_AAEL002761|tropomyosin 1;K10374|5|3e-22|103|ame:408414|tropomyosin 2 | - | - | - | gi|114052272|ref|NP_001040465.1|/5.92587e-37/tropomyosin-2 isoform 2 [Bombyx mori] |
| 61 | Bm_nscaf2767_131 | 498 |  |  |  | 10.3 |  | - | - | - | - | gi|379698926|ref|NP_001243934.1|/4.75482e-68/putative cuticle protein CPH45 precursor [Bombyx mori] |
| 62 | Bm_nscaf2839_21 | 465 |  |  |  | 9.6 |  | K09443|1|5e-35|146|nvi:100119224|ETS-type family, other;K09437|2|6e-35|146|mdo:100010747|ETS oncogene family protein FEV | GO:0043231//intracellular membrane-bounded organelle | GO:0003712//transcription cofactor activity;GO:0001071//nucleic acid binding transcription factor activity;GO:0043566//structure-specific DNA binding | GO:0048869//cellular developmental process;GO:0048731;GO:0006355//regulation of transcription, DNA-dependent | gi|357620574|gb|EHJ72723.1|/1.55698e-45/hypothetical protein KGM_16097 [Danaus plexippus] |
| 63 | Bm_nscaf2767_136 | 561 |  |  |  | 9.2 |  | - | - | - | - | gi|262034617|gb|ACY06933.1|/6.41675e-18/putative cuticle protein CPH38 [Bombyx mori] |
| 64 | Bm_nscaf463_08 | 1062 |  |  |  | 9.2 |  | K12373|1|6e-97|354|cqu:CpipJ_CPIJ016910|hexosaminidase [EC:3.2.1.52] | - | GO:0015929//hexosaminidase activity;GO:0043167//ion binding | GO:0044238//primary metabolic process | gi|112982942|ref|NP_001037096.1|/0/beta-N-acetylglucosaminidase 3 precursor [Bombyx mori] |
| 65 | Bm_nscaf3003_091 | 1524 |  |  |  | 8.0 |  | K01229|1|5e-138|491|aag:AaeL_AAEL009246|lactase-phlorizin hydrolase [EC:3.2.1.108 3.2.1.62] | - | GO:0016787//hydrolase activity | - | gi|2970687|gb|AAC06038.1|/0/beta-glucosidase precursor [Spodoptera frugiperda] |
| 66 | Bm_nscaf2575_007 | 2673 |  |  |  | 7.7 |  | - | - | - | - | gi|357625335|gb|EHJ75815.1|/6.78832e-96/hypothetical protein KGM_07589 [Danaus plexippus] |
| 67 | Bm_nscaf2865_028 | 3318 |  |  |  | 7.5 |  | K00430|1|1e-75|285|cel:F09F3.5|peroxidase [EC:1.11.1.7];K10789|4|3e-71|271|aml:100472895|myeloperoxidase [EC:1.11.1.7] | - | - | - | gi|347972907|ref|XP_317106.5|/0/AGAP008350-PA [Anopheles gambiae str. PEST] |
| 68 | Bm_nscaf98_59 | 3528 |  |  |  | 6.9 |  | K10352|1|0.0|1232|ame:409843|myosin heavy chain | GO:0016459//myosin complex | GO:0008092//cytoskeletal protein binding;GO:0017111//nucleoside-triphosphatase activity;GO:0032559 | - | gi|312370897|gb|EFR19200.1|/0/hypothetical protein AND_22908 [Anopheles darlingi] |
| 69 | Bm_nscaf2210_072 | 10284 |  |  |  | 5.8 |  | K00907|1|9e-148|527|dya:Dyak_GE14115|myosin-light-chain kinase [EC:2.7.11.18] | - | GO:0016301//kinase activity | - | gi|357622614|gb|EHJ74040.1|/0/Stretchin-Mlck, isoform A [Danaus plexippus] |
| 70 | Bm_nscaf56_1 | 870 |  |  |  | 4.7 | 4.0 | - | - | - | - | gi|389611748|dbj|BAM19454.1|/1.0678e-21/unknown secreted protein, partial [Papilio xuthus] |
| 71 | Bm_nscaf2330_060 | 747 |  |  |  | 4.5 |  | K07739|1|5e-21|101|dan:Dana_GF21222|elongator complex protein 3 [EC:2.3.1.48] | - | - | - | gi|389609417|dbj|BAM18320.1|/7.4702e-44/similar to CG10527 [Papilio xuthus] |
| 72 | Bm_nscaf2998_74 | 1062 |  |  |  | 4.4 |  | K00507|1|4e-129|461|dan:Dana_GF17961|stearoyl-CoA desaturase (delta-9 desaturase) [EC:1.14.19.1] | GO:0043231//intracellular membrane-bounded organelle;GO:0031224//intrinsic to membrane | GO:0016215;GO:0046914//transition metal ion binding | GO:0006631//fatty acid metabolic process | gi|112983214|ref|NP_001037018.1|/0/acyl-CoA desaturase [Bombyx mori] |
| 73 | Bm_nscaf2868_56 | 1860 |  |  |  | 4.4 |  | K01187|1|1e-162|573|dme:Dmel_CG11909|alpha-glucosidase [EC:3.2.1.20];K05546|2|9e-20|99.4|smm:Smp_018760|alpha 1,3-glucosidase [EC:3.2.1.84];K12317|4|1e-18|95.9|aml:100469731|neutral alpha-glucosidase C [EC:3.2.1.20] | - | - | - | gi|103058158|gb|ABF71570.1|/0/glycosyl hydrolase family 31 protein [Bombyx mori] |
| 74 | Bm_nscaf3005_56 | 1479 |  |  |  | 4.2 |  | K07424|1|3e-78|293|ame:412209|cytochrome P450, family 3, subfamily A [EC:1.14.14.1];K15002|2|2e-74|280|aag:AaeL_AAEL014678|cytochrome P450, family 6 [EC:1.14.-.-] | - | GO:0046872//metal ion binding | - | gi|291464095|gb|ADE05585.1|/7.4103e-177/cytochrome P450 6AN5 [Manduca sexta] |
| 75 | Bm_nscaf1962_13 | 816 |  |  |  | 4.0 |  | K01312|1|3e-29|129|tca:655678|trypsin [EC:3.4.21.4] | - | - | - | gi|282721218|gb|ADA83702.1|/3.3086e-83/trypsin [Helicoverpa armigera] |
| 76 | Bm_nscaf2902_244 | 1776 |  |  |  | 3.9 |  | K01904|1|3e-57|223|cel:ZK1127.2|4-coumarate--CoA ligase [EC:6.2.1.12];K00666|2|4e-46|186|mmu:264895|fatty-acyl-CoA synthase [EC:6.2.1.-] | - | GO:0003824//catalytic activity | - | gi|357605939|gb|EHJ64841.1|/0/putative AMP dependent coa ligase [Danaus plexippus] |
| 77 | Bm_nscaf2674_089 | 531 |  |  |  | 3.6 |  | - | - | - | - | gi|357609390|gb|EHJ66426.1|/1.2182e-49/hypothetical protein KGM_10793 [Danaus plexippus] |
| 78 | Bm_nscaf2888_085 | 1563 |  |  |  | 3.5 |  | K07763|1|0.0|684|dpo:Dpse_GA18484|matrix metalloproteinase-14 (membrane-inserted) [EC:3.4.24.80];K08006|2|2e-89|330|bfo:BRAFLDRAFT_230518|matrix metalloproteinase-28 (epilysin) [EC:3.4.24.-];K08002|3|3e-89|329|spu:574861|matrix metalloproteinase-24 (membrane-inserted) [EC:3.4.24.-] | GO:0043005//neuron projection;GO:0044421//extracellular region part | GO:0004175//endopeptidase activity;GO:0046914//transition metal ion binding | GO:0009888//tissue development;GO:0035151//regulation of tube size, open tracheal system;GO:0022617//extracellular matrix disassembly;GO:0002164//larval development;GO:0019538//protein metabolic process;GO:0048102//autophagic cell death;GO:0007560//imaginal disc morphogenesis | gi|172356113|ref|NP_001116499.1|/0/matrix metalloproteinase 1 isoform 1 [Bombyx mori] |
| 79 | Bm_nscaf2674_120 | 2406 |  |  |  | 3.5 |  | K05316|1|0.0|783|tca:658471|voltage-dependent calcium channel alpha-2/delta, invertebrate | - | - | - | gi|357606470|gb|EHJ65081.1|/0/putative voltage-gated calcium channel alpha2-delta subunit 1 [Danaus plexippus] |
| 80 | Bm_nscaf2951_26 | 1584 |  |  |  | 3.5 |  | - | - | - | - | gi|357614606|gb|EHJ69170.1|/1.12117e-85/hypothetical protein KGM_15717 [Danaus plexippus] |
| 81 | Bm_nscaf2865_185 | 1350 |  |  |  | 3.4 |  | K04308|1|9e-24|112|bta:520189|leucine-rich repeat-containing G protein-coupled receptor 5 | - | - | - | gi|357603260|gb|EHJ63681.1|/1.05745e-142/hypothetical protein KGM_06598 [Danaus plexippus] |
| 82 | Bm_nscaf2943_045 | 3249 |  |  |  | 3.3 |  | K05328|1|0.0|984|aag:AaeL_AAEL005437|transient receptor potential cation channel subfamily C, invertebrate | - | - | GO:0006810//transport | gi|357607163|gb|EHJ65374.1|/0/transient receptor potential channel [Danaus plexippus] |
| 83 | Bm_nscaf2511_147 | 1929 |  |  |  | 3.3 |  | K13046|1|0.0|847|cqu:CpipJ_CPIJ012475|tolkin [EC:3.4.24.-] | - | GO:0046872//metal ion binding;GO:0008233//peptidase activity | - | gi|332021857|gb|EGI62193.1|/0/Tolloid-like protein 2 [Acromyrmex echinatior] |
| 84 | Bm_nscaf2825_04 | 228 |  |  |  | 3.1 |  | - | GO:0016020//membrane | GO:0017111//nucleoside-triphosphatase activity;GO:0000166//nucleotide binding | - | gi|357610565|gb|EHJ67040.1|/5.56389e-19/hypothetical protein KGM_03614 [Danaus plexippus] |
| 85 | Bm_nscaf1898_501 | 1095 |  |  |  | 2.9 |  | K09442|1|5e-43|175|tca:656847|SAM pointed domain-containing ETS transcription factor | - | - | - | gi|357628928|gb|EHJ78027.1|/5.39486e-168/hypothetical protein KGM_21536 [Danaus plexippus] |
| 86 | Bm_nscaf2865_137 | 1299 |  |  |  | 2.9 |  | K03103|1|3e-44|180|ame:725931|multiple inositol-polyphosphate phosphatase [EC:3.1.3.62] | - | GO:0016791//phosphatase activity | - | gi|357623927|gb|EHJ74885.1|/1.26548e-137/putative multiple inositol polyphosphate phosphatase [Danaus plexippus] |
| 87 | Bm_nscaf3079_28 | 714 |  |  |  | 2.8 |  | K08768|1|2e-06|53.1|pon:100437952|perilipin | - | - | - | gi|114051003|ref|NP_001040143.1|/2.42157e-73/perilipin [Bombyx mori] |
| 88 | Bm_nscaf2511_145 | 1644 |  |  |  | 2.8 |  | K13046|1|2e-69|264|ame:410386|tolkin [EC:3.4.24.-] | GO:0044464//cell part | GO:0004175//endopeptidase activity;GO:0046914//transition metal ion binding | GO:0048731;GO:0019538//protein metabolic process;GO:0009653//anatomical structure morphogenesis | gi|340722615|ref|XP_003399699.1|/8.19946e-71/PREDICTED: tolloid-like protein 2-like [Bombus terrestris] |
| 89 | Bm_nscaf2789_62 | 1134 |  |  |  | 2.7 | 3.6 | K01915|1|5e-125|447|tca:656087|glutamine synthetase [EC:6.3.1.2] | GO:0044424//intracellular part | GO:0016211//ammonia ligase activity;GO:0032559 | GO:0043112//receptor metabolic process;GO:0006541//glutamine metabolic process;GO:0007399//nervous system development;GO:0006536//glutamate metabolic process | gi|389608625|dbj|BAM17922.1|/6.74547e-161/glutamine synthetase 2 [Papilio xuthus] |
| 90 | Bm_nscaf2674_128 | 4341 |  |  |  | 2.7 |  | K04527|1|0.0|993|tca:661524|insulin receptor [EC:2.7.10.1] | GO:0031224//intrinsic to membrane | GO:0004871//signal transducer activity;GO:0004713//protein tyrosine kinase activity;GO:0032559;GO:0019900//kinase binding | GO:0006468//protein phosphorylation;GO:0007167//enzyme linked receptor protein signaling pathway | gi|112983268|ref|NP_001037011.1|/0/insulin receptor precursor [Bombyx mori] |
| 91 | Bm_nscaf3055_38 | 645 |  |  |  | 2.7 |  | - | - | - | - | gi|357613968|gb|EHJ68817.1|/3.39697e-41/hypothetical protein KGM_13715 [Danaus plexippus] |
| 92 | Bm_nscaf2888_446 | 1821 |  |  |  | 2.6 |  | K02599|1|2e-47|191|dre:794892|Notch | GO:0044459//plasma membrane part | GO:0005488//binding | GO:0001745//compound eye morphogenesis;GO:0071842;GO:0002009//morphogenesis of an epithelium;GO:0035088//establishment or maintenance of apical/basal cell polarity;GO:0030154//cell differentiation;GO:0050794//regulation of cellular process;GO:0007399//ner | gi|357620129|gb|EHJ72435.1|/0/crumbs [Danaus plexippus] |
| 93 | Bm_nscaf3033_09 | 444 |  |  |  | 2.6 |  | K00791|1|6e-29|126|tca:659205|tRNA dimethylallyltransferase [EC:2.5.1.75] | GO:0043231//intracellular membrane-bounded organelle | - | - | gi|357621626|gb|EHJ73401.1|/5.31779e-38/hypothetical protein KGM_06387 [Danaus plexippus] |
| 94 | Bm_nscaf2825_03 | 1863 |  |  |  | 2.6 |  | K05679|1|1e-133|477|dre:556979|ATP-binding cassette, subfamily G (WHITE), member 1;K05680|2|3e-133|476|cin:100179107|ATP-binding cassette, subfamily G (WHITE), member 4 | GO:0016020//membrane | GO:0017111//nucleoside-triphosphatase activity;GO:0000166//nucleotide binding | - | gi|326371147|gb|ADZ56942.1|/0/ATP-binding cassette transporter subfamily G [Bombyx mori] |
| 95 | Bm_nscaf2943_026 | 3330 |  |  |  | 2.6 |  | - | - | - | - | gi|357625119|gb|EHJ75661.1|/0/hypothetical protein KGM_14730 [Danaus plexippus] |
| 96 | Bm_nscaf3026_228 | 1458 |  |  |  | 2.6 |  | K13356|1|1e-163|577|dvi:Dvir_GJ20993|fatty acyl-CoA reductase [EC:1.2.1.-] | - | - | - | gi|357619249|gb|EHJ71899.1|/0/hypothetical protein KGM_16194 [Danaus plexippus] |
| 97 | Bm_nscaf2829_162 | 573 |  |  |  | 2.4 |  | - | - | - | - | gi|357617004|gb|EHJ70527.1|/1.46568e-28/hypothetical protein KGM_07241 [Danaus plexippus] |
| 98 | Bm_nscaf2575_181 | 3174 |  |  |  | 2.4 |  | K13253|1|0.0|1165|tca:655549|nitric-oxide synthase, invertebrate [EC:1.14.13.39] | - | GO:0016709//oxidoreductase activity, acting on paired donors, with incorporation or reduction of molecular oxygen, NADH or NADPH as one donor, and incorporation of one atom of oxygen;GO:0005506//iron ion binding;GO:0005515//protein binding;GO:0050662//coe | GO:0044271//cellular nitrogen compound biosynthetic process | gi|112983340|ref|NP_001036963.1|/0/nitric oxide synthase [Bombyx mori] |
| 99 | Bm_nscaf2770_58 | 684 |  |  |  | 2.3 |  | K09268|1|2e-31|135|ame:726914|transcription factor SOX4/11/12 (SOX group C) | - | GO:0003676//nucleic acid binding;GO:0016491//oxidoreductase activity | - | gi|357605025|gb|EHJ64439.1|/1.35368e-70/putative transcription factor SOX-14 [Danaus plexippus] |
| 100 | Bm_nscaf2575_006 | 840 |  |  |  | 2.3 |  | - | - | - | - | gi|357625337|gb|EHJ75817.1|/7.89516e-35/hypothetical protein KGM_07577 [Danaus plexippus] |
| 101 | Bm_nscaf2865_051 | 156 |  |  |  | 2.3 |  | - | - | - | GO:0035264//multicellular organism growth | gi|357626410|gb|EHJ76511.1|/1.66372e-10/putative Cytohesin-1 [Danaus plexippus] |
| 102 | Bm_nscaf2330_003 | 732 |  |  |  | 2.2 |  | K06560|1|2e-19|96.3|bfo:BRAFLDRAFT_80311|mannose receptor, C type | - | GO:0030246//carbohydrate binding | - | gi|284813581|ref|NP_001165396.1|/1.62104e-144/C-type lectin 19 precursor [Bombyx mori] |
| 103 | Bm_nscaf2204_084 | 255 |  |  |  | 2.2 |  | - | - | GO:0016655//oxidoreductase activity, acting on NADH or NADPH, quinone or similar compound as acceptor;GO:0046914//transition metal ion binding | GO:0008152//metabolic process | gi|357614619|gb|EHJ69177.1|/3.53817e-13/vesicle amine transport protein [Danaus plexippus] |
| 104 | Bm_nscaf2902_301 | 384 |  |  |  | 2.2 |  | - | - | GO:0004091//carboxylesterase activity | - | gi|357623142|gb|EHJ74409.1|/1.75071e-65/putative tubulin-specific chaperone e [Danaus plexippus] |
| 105 | Bm_nscaf3079_27 | 714 |  |  |  | 2.2 |  | - | - | - | - | gi|114051003|ref|NP_001040143.1|/6.96445e-113/perilipin [Bombyx mori] |
| 106 | Bm_nscaf2902_300 | 948 |  |  |  | 2.1 |  | K10160|1|5e-06|52.4|tgu:100228273|toll-like receptor 4 | - | - | - | gi|357623142|gb|EHJ74409.1|/3.35625e-128/putative tubulin-specific chaperone e [Danaus plexippus] |
| 107 | Bm_nscaf2951_32 | 1782 |  |  |  | 2.1 |  | K00311|1|0.0|857|nvi:100119918|electron-transferring-flavoprotein dehydrogenase [EC:1.5.5.1] | GO:0019866//organelle inner membrane;GO:0044444//cytoplasmic part;GO:0015630//microtubule cytoskeleton | GO:0016491//oxidoreductase activity | GO:0006091//generation of precursor metabolites and energy | gi|357614610|gb|EHJ69174.1|/0/hypothetical protein KGM_15720 [Danaus plexippus] |
| 108 | Bm_nscaf2655_005 | 1767 |  |  |  | 2.1 |  | K11583|1|4e-157|555|tca:658209|protein phosphatase 2 (formerly 2A), regulatory subunit B'' | - | GO:0046872//metal ion binding | - | gi|357617009|gb|EHJ70530.1|/0/hypothetical protein KGM_09283 [Danaus plexippus] |
| 109 | Bm_nscaf3068_11 | 318 |  |  |  | 2.1 |  | - | - | - | - | gi|357618575|gb|EHJ71507.1|/1.00267e-20/endonuclease-reverse transcriptase HmRTE-e01 [Danaus plexippus] |
| 110 | Bm_nscaf2876_58 | 1407 |  |  |  | 2.0 |  | - | - | - | - | gi|379046450|gb|AFC87786.1|/0/yellow-d [Bombyx mori] |
| 111 | Bm_nscaf3068_12 | 2337 |  |  |  | 2.0 |  | K08765|1|0.0|1064|nvi:100121693|carnitine O-palmitoyltransferase 1 [EC:2.3.1.21] | GO:0043231//intracellular membrane-bounded organelle | GO:0016406 | GO:0010035//response to inorganic substance;GO:0006950//response to stress | gi|357605446|gb|EHJ64634.1|/0/putative carnitine o-acyltransferase [Danaus plexippus] |
| 112 | Bm_nscaf3058_122 | 267 |  |  |  | 2.0 |  | - | - | - | - | gi|357631631|gb|EHJ79100.1|/8.87561e-25/hypothetical protein KGM_15577 [Danaus plexippus] |
| 113 | Bm_nscaf3026_073 | 915 |  |  |  | 2.0 |  | K01053|1|3e-61|235|nvi:100113493|gluconolactonase [EC:3.1.1.17] | - | - | - | gi|389610187|dbj|BAM18705.1|/1.08242e-136/regucalcin [Papilio xuthus] |
| 114 | Bm_nscaf2809_14 | 1467 |  |  |  | -2.0 |  | K02375|1|9e-155|547|xtr:100488110|frizzled 5/8 | GO:0031224//intrinsic to membrane | GO:0004888//transmembrane signaling receptor activity | GO:0050793//regulation of developmental process;GO:0048468//cell development;GO:0008283//cell proliferation;GO:0007166//cell surface receptor signaling pathway;GO:0001654//eye development | gi|350408848|ref|XP_003488535.1|/3.17834e-180/PREDICTED: frizzled-2-like [Bombus impatiens] |
| 115 | Bm_nscaf2766_13 | 2181 |  |  |  | -2.0 |  | K11810|1|2e-40|167|xtr:394932|MFS transporter, MCP family, solute carrier family 16 (monocarboxylic acid transporters), member 12 | - | - | - | gi|357607867|gb|EHJ65719.1|/0/putative monocarboxylate transporter [Danaus plexippus] |
| 116 | Bm_nscaf2529_073 | 507 |  |  |  | -2.0 |  | - | - | - | - | gi|389608893|dbj|BAM18058.1|/8.14648e-50/similar to CG2765 [Papilio xuthus] |
| 117 | Bm_nscaf2674_296 | 996 |  |  |  | -2.1 |  | K05324|1|2e-48|193|tca:660459|potassium voltage-gated channel KQT-like subfamily, invertebrate | GO:0005887//integral to plasma membrane | GO:0005267//potassium channel activity | GO:0006941//striated muscle contraction;GO:0008016//regulation of heart contraction;GO:0034220//ion transmembrane transport;GO:0009792//embryo development ending in birth or egg hatching;GO:0030001//metal ion transport | gi|357628260|gb|EHJ77650.1|/3.49371e-131/hypothetical protein KGM_04614 [Danaus plexippus] |
| 118 | Bm_nscaf2589_179 | 1047 |  |  |  | -2.3 |  | K09228|1|4e-60|232|api:100161299|KRAB domain-containing zinc finger protein | GO:0044464//cell part | GO:0046914//transition metal ion binding | GO:0009653//anatomical structure morphogenesis;GO:0002165//instar larval or pupal development | gi|295424086|ref|NP_001171332.1|/0/Kruppel homolog 1 [Bombyx mori] |
| 119 | Bm_nscaf3090_4 | 834 |  |  |  | -2.3 |  | K10052|1|8e-37|154|aag:AaeL_AAEL002853|CCAAT/enhancer binding protein (C/EBP), invertebrate | GO:0043231//intracellular membrane-bounded organelle | GO:0003677//DNA binding;GO:0001071//nucleic acid binding transcription factor activity;GO:0005515//protein binding | GO:0006351//transcription, DNA-dependent | gi|112983730|ref|NP_001037374.1|/2.88559e-122/chorion specific C/EBP [Bombyx mori] |
| 120 | Bm_scaffold944_1 | 1161 |  |  |  | -3.0 |  | K03283|1|7e-130|464|dya:Dyak_GE26149|heat shock 70kDa protein 1/8 | - | GO:0032559 | GO:0050896//response to stimulus | gi|195500279|ref|XP_002097305.1|/5.36384e-129/GE26149 [Drosophila yakuba] |
| 121 | Bm_nscaf2801_19 | 1668 |  |  |  | -3.2 |  | K03283|1|0.0|664|tca:100142517|heat shock 70kDa protein 1/8 | - | GO:0032559 | GO:0050896//response to stimulus | gi|357622391|gb|EHJ73892.1|/0/HSP70 [Danaus plexippus] |
| 122 | Bm_nscaf2993_259 | 1563 |  |  |  | -3.2 |  | - | - | - | - | gi|357602928|gb|EHJ63570.1|/2.83932e-41/hypothetical protein KGM_12716 [Danaus plexippus] |
| 123 | Bm_nscaf2891_057 | 525 |  |  |  | -3.3 |  | K11147|1|4e-16|84.3|dgr:Dgri_GH15276|dehydrogenase/reductase SDR family member 4 [EC:1.1.-.-];K00081|2|6e-15|80.5|ssc:396780|carbonyl reductase 2 [EC:1.1.1.184] | - | - | - | gi|328782074|ref|XP_394891.4|/3.54847e-30/PREDICTED: tetratricopeptide repeat protein 27-like [Apis mellifera] |
| 124 | Bm_nscaf2801_20 | 762 |  |  |  | -3.7 |  | K03283|1|9e-69|260|tca:100142517|heat shock 70kDa protein 1/8 | - | GO:0032559 | GO:0050896//response to stimulus | gi|397523644|ref|XP_003831834.1|/1.60755e-73/PREDICTED: heat shock 70 kDa protein 1A/1B-like [Pan paniscus] |
| 125 | Bm_nscaf3076_4 | 504 |  |  |  | -4.0 |  | K09542|1|1e-12|72.8|bfo:BRAFLDRAFT_130908|crystallin, alpha B | - | - | GO:0006950//response to stress | gi|283483979|ref|NP_001164470.1|/2.89404e-92/19.5 kDa heat shock protein [Bombyx mori] |
| 126 | Bm_nscaf1898_327 | 1620 |  |  |  | -4.4 |  | K13358|1|1e-09|65.5|cin:100175118|Na(+)/H(+) exchange regulatory cofactor NHE-RF2;K13365|2|1e-08|62.4|dre:100329719|Na(+)/H(+) exchange regulatory cofactor NHE-RF1;K08018|5|3e-07|57.8|bfo:BRAFLDRAFT_131151|Rap guanine nucleotide exchange factor (GEF) 2 | - | - | - | gi|91081527|ref|XP_974840.1|/1.61998e-71/PREDICTED: similar to harmonin [Tribolium castaneum] |
| 127 | Bm_nscaf2818_106 | 759 |  |  |  |  | 4.5 | K13187|1|9e-15|80.9|bfo:BRAFLDRAFT_83361|RNA-binding protein 4 | - | GO:0005488//binding | - | gi|114053173|ref|NP_001040506.1|/4.13856e-122/bmp-2 protein [Bombyx mori] |
| 128 | Bm_nscaf2931_28 | 243 |  |  |  |  | 3.1 | - | - | - | - | gi|112983466|ref|NP_001037532.1|/1.3091e-12/fungal protease inhibitor F precursor [Bombyx mori] |
| 129 | Bm_nscaf2853_172 | 264 |  |  |  |  | 2.4 | - | - | - | - | - |
| 130 | Bm_nscaf463_19 | 1170 |  |  |  |  | -2.2 | K04153|1|1e-123|443|tca:655795|5-hydroxytryptamine receptor 1 | GO:0016021//integral to membrane | GO:0004993//serotonin receptor activity | GO:0007200//phospholipase C-activating G-protein coupled receptor signaling pathway;GO:0007193//adenylate cyclase-inhibiting G-protein coupled receptor signaling pathway;GO:0009605//response to external stimulus | gi|113203435|gb|ABI33826.1|/0/putative serotonin receptor [Manduca sexta] |
| 131 | Bm_nscaf3079_51 | 1575 |  |  |  |  | -2.4 | K06258|1|1e-68|261|aag:AaeL_AAEL012098|MFS transporter, VNT family, synaptic vesicle glycoprotein 2 | - | - | - | gi|357616637|gb|EHJ70296.1|/0/SV2-like protein 1 [Danaus plexippus] |
| 132 | Bm_nscaf3045_59 | 723 |  |  |  |  | -3.0 | K01135|1|4e-30|131|dme:Dmel_CG8646|arylsulfatase B [EC:3.1.6.12];K12375|4|4e-18|92.0|xtr:100494972|arylsulfatase I/J [EC:3.1.6.-] | - | - | - | gi|22450117|emb|CAC86342.1|/3.50352e-43/glucosinolate sulfatase [Plutella xylostella] |
| Total | | up | 10 | 20 | 18 | 74 | 8 |  |  |  |  |  |
|  |  | down | 0 | 1 | 8 | 13 | 3 |  |  |  |  |  |
