# Supplementary material for: Gene Expression Profile of Bombyx mori Hemocyte under the Stress of Destruxin A
Source: PLoS One. 2014 May 6;9(5):e96170. doi: 10.1371/journal.pone.0096170 (PMC4011735; doi:10.1371/journal.pone.0096170)
Supplement: Table S2 — Details of KEGG pathway enrichment analysis of the genes with significant expression change. (DOC) [file pone.0096170.s006.doc]

Supplementary file 6 Pathways of genes with the expression more than 2-fold changes

| # | Pathway | 1 h | 4 h | 8 h | 12 h | 24 h |
| --- | --- | --- | --- | --- | --- | --- |
| 1 | ABC transporters |  |  |  | Bm_nscaf2825_03 |  |
| 2 | Acute myeloid leukemia |  | Bm_nscaf2136_098 |  |  |  |
| 3 | Adherens junction |  | Bm_nscaf3099_002 |  | Bm_nscaf2674_128 |  |
| 4 | Adipocytokine signaling pathway |  |  |  | Bm_nscaf3068_12 |  |
| 5 | African trypanosomiasis |  |  |  |  |  |
| 6 | Alanine, aspartate and glutamate metabolism |  |  |  | Bm_nscaf2789_62 | Bm_nscaf2789_62 |
| 7 | Alcoholism |  | Bm_nscaf2136_098 |  |  |  |
| 8 | Aldosterone-regulated sodium reabsorption |  |  |  | Bm_nscaf2674_128, Bm_nscaf1898_327 |  |
| 9 | alpha-Linolenic acid metabolism |  |  | Bm_nscaf2529_052 |  |  |
| 10 | Amino sugar and nucleotide sugar metabolism |  |  |  | Bm_nscaf463_08 |  |
| 11 | Amoebiasis |  | Bm_nscaf3031_265, Bm_nscaf2888_193 |  | Bm_nscaf3031_265, Bm_nscaf2902_300 |  |
| 12 | Antigen processing and presentation |  |  | Bm_nscaf2795_018 | Bm_nscaf2801_20, Bm_nscaf2801_19, Bm_scaffold944_1 |  |
| 13 | Arachidonic acid metabolism |  |  |  | Bm_nscaf2891_057 |  |
| 14 | Arginine and proline metabolism |  |  |  | Bm_nscaf2789_62, Bm_nscaf2575_181 | Bm_nscaf2789_62 |
| 15 | Arrhythmogenic right ventricular cardiomyopathy (ARVC) |  | Bm_nscaf3099_002 |  |  |  |
| 16 | Axon guidance |  | Bm_nscaf2136_098 |  |  |  |
| 17 | B cell receptor signaling pathway |  | Bm_nscaf2136_098 |  |  |  |
| 18 | Bacterial invasion of epithelial cells |  | Bm_nscaf3099_002 |  |  |  |
| 19 | Bile secretion |  |  |  | Bm_nscaf3005_56 |  |
| 20 | Biosynthesis of unsaturated fatty acids |  |  |  | Bm_nscaf2998_74, |  |
| 21 | Calcium signaling pathway |  |  |  | Bm_nscaf2210_072 |  |
| 22 | Carbohydrate digestion and absorption |  |  | Bm_nscaf2855_239 | Bm_nscaf3003_091 |  |
| 23 | Cardiac muscle contraction |  |  | Bm_nscaf2930_138 | Bm_nscaf2136_210, Bm_nscaf98_59 |  |
| 24 | Cell adhesion molecules (CAMs) |  |  |  |  |  |
| 25 | Chagas disease (American trypanosomiasis) |  |  |  | Bm_nscaf2902_300 |  |
| 26 | Chemokine signaling pathway |  | Bm_nscaf2136_098 |  |  |  |
| 27 | Chronic myeloid leukemia |  | Bm_nscaf2136_098 |  |  |  |
| 28 | Circadian rhythm - fly |  |  |  | Bm_nscaf3072_46 |  |
| 29 | Collecting duct acid secretion | Bm_nscaf2889_046 |  |  |  |  |
| 30 | Dilated cardiomyopathy |  | Bm_nscaf3099_002 | Bm_nscaf2930_138 | Bm_nscaf2136_210, Bm_nscaf98_59 |  |
| 31 | Dopaminergic synapse |  |  |  | Bm_nscaf2655_005 |  |
| 32 | Dorso-ventral axis formation |  | Bm_nscaf2136_098 |  | Bm_nscaf2888_446 |  |
| 33 | Drug metabolism - cytochrome P450 |  |  |  | Bm_nscaf3005_56 |  |
| 34 | Drug metabolism - other enzymes |  |  |  | Bm_nscaf3005_56, |  |
| 35 | ECM-receptor interaction |  | Bm_nscaf3031_265, Bm_nscaf2888_193 | Bm_scaffold416_04 | Bm_nscaf3031_265 | Bm_nscaf3079_51 |
| 36 | Endocytosis |  |  |  | Bm_nscaf2801_20, Bm_nscaf2801_19, Bm_scaffold944_1 |  |
| 37 | Endometrial cancer |  | Bm_nscaf2136_098 |  |  |  |
| 38 | Epstein-Barr virus infection |  |  |  | Bm_nscaf2801_20, Bm_nscaf2801_19, Bm_scaffold944_1 |  |
| 39 | ErbB signaling pathway |  | Bm_nscaf2136_098 |  |  |  |
| 40 | Fanconi anemia pathway |  |  | Bm_nscaf2855_239 |  |  |
| 41 | Fat digestion and absorption |  |  | Bm_nscaf2529_052, Bm_nscaf3035_065 |  |  |
| 42 | Fatty acid metabolism | Bm_nscaf2674_064, Bm_nscaf2674_063, Bm_nscaf2983_049, Bm_nscaf2674_066 |  |  | Bm_nscaf3068_12 |  |
| 43 | Fc epsilon RI signaling pathway |  | Bm_nscaf2136_098 |  |  |  |
| 44 | Focal adhesion | Bm_nscaf2674_064, Bm_nscaf2674_063, Bm_nscaf2983_049, Bm_nscaf2674_066 |  |  | Bm_nscaf2210_072 |  |
| 45 | Fructose and mannose metabolism |  |  |  |  | Bm_nscaf3035_060 |
| 46 | GABAergic synapse |  |  |  | Bm_nscaf2789_62 | Bm_nscaf2789_62 |
| 47 | Galactose metabolism |  | Bm_nscaf2136_098, Bm_nscaf3031_265, Bm_nscaf2888_193, Bm_nscaf3099_002, | Bm_nscaf2855_239 | Bm_nscaf3003_091, Bm_nscaf2868_56 | Bm_nscaf3035_060 |
| 48 | Gap junction |  | Bm_nscaf2136_098 |  |  |  |
| 49 | Gastric acid secretion |  | Bm_nscaf3099_002 |  | Bm_nscaf2210_072 |  |
| 50 | Glioma |  | Bm_nscaf2136_098 |  |  |  |
| 51 | Glutamatergic synapse |  |  |  | Bm_nscaf2789_62 | Bm_nscaf2789_62 |
| 52 | Glutathione metabolism | Bm_nscaf2889_046 |  |  |  |  |
| 53 | Glycerolipid metabolism |  |  | Bm_nscaf2529_052, Bm_nscaf3035_065 |  | Bm_nscaf3035_060 |
| 54 | Glycerophospholipid metabolism |  |  | Bm_nscaf2529_052 |  |  |
| 55 | Glycine, serine and threonine metabolism |  |  | Bm_nscaf2847_194 |  |  |
| 56 | Glycosaminoglycan degradation |  |  |  | Bm_nscaf463_08 | Bm_nscaf3045_59 |
| 57 | Glycosphingolipid biosynthesis - ganglio series |  |  |  | Bm_nscaf463_08 |  |
| 58 | Glycosphingolipid biosynthesis - globo series |  |  |  | Bm_nscaf463_08 |  |
| 59 | Glyoxylate and dicarboxylate metabolism |  |  |  | Bm_nscaf2789_62 | Bm_nscaf2789_62 |
| 60 | GnRH signaling pathway |  | Bm_nscaf2136_098 |  | Bm_nscaf2888_085 |  |
| 61 | Hematopoietic cell lineage | Bm_nscaf2889_046 |  | Bm_scaffold416_04 |  |  |
| 62 | Hepatitis C |  | Bm_nscaf2136_098 |  |  |  |
| 63 | Homologous recombination |  |  | Bm_nscaf2855_239 |  |  |
| 64 | Huntington's disease |  | Bm_nscaf2589_196 |  | Bm_nscaf2589_196, |  |
| 65 | Hypertrophic cardiomyopathy (HCM) |  | Bm_nscaf3099_002 | Bm_nscaf2930_138 | Bm_nscaf2136_210, Bm_nscaf98_59 |  |
| 66 | Influenza A | Bm_nscaf2674_064, Bm_nscaf2674_063, Bm_nscaf2983_049, Bm_nscaf2674_066, trypsin, actin | Bm_nscaf3099_002 | Bm_nscaf2983_049, Bm_nscaf2674_066 | Bm_nscaf1962_13, Bm_nscaf2983_049, Bm_nscaf2902_300, Bm_nscaf2801_20, Bm_nscaf2801_19, Bm_scaffold944_1 |  |
| 67 | Inositol phosphate metabolism |  |  |  | Bm_nscaf2865_137 |  |
| 68 | Insulin signaling pathway |  | Bm_nscaf2136_098 |  | Bm_nscaf2674_128 |  |
| 69 | Jak-STAT signaling pathway |  | Bm_nscaf2136_098 |  |  |  |
| 70 | Legionellosis |  |  |  | Bm_nscaf2902_300, Bm_nscaf2801_20, Bm_nscaf2801_19, Bm_scaffold944_1 |  |
| 71 | Leishmaniasis |  |  |  | Bm_nscaf2902_300 |  |
| 72 | Leukocyte transendothelial migration |  | Bm_nscaf3099_002 |  |  |  |
| 73 | Linoleic acid metabolism |  |  |  | Bm_nscaf3005_56 |  |
| 74 | Lysine degradation |  |  | Bm_nscaf2930_138 |  |  |
| 75 | Lysosome |  |  | Bm_nscaf2795_018 | Bm_nscaf463_08 | Bm_nscaf3045_59 |
| 76 | Malaria |  |  |  | Bm_nscaf2902_300 |  |
| 77 | MAPK signaling pathway |  | Bm_nscaf2136_098, Bm_nscaf2888_274, |  | Bm_nscaf2674_120, Bm_nscaf1898_327, Bm_nscaf2801_20, Bm_nscaf2801_19, Bm_scaffold944_1 |  |
| 78 | MAPK signaling pathway - fly |  | Bm_nscaf2136_098 |  |  |  |
| 79 | Measles |  |  |  | Bm_nscaf2902_300, Bm_nscaf2801_20, Bm_nscaf2801_19, Bm_scaffold944_1 |  |
| 80 | Melanogenesis |  |  | Bm_nscaf2930_184 |  |  |
| 81 | Metabolic pathways | Bm_nscaf2889_046 |  | Bm_nscaf2529_052, Bm_nscaf2930_184, Bm_nscaf2855_23 | Bm_nscaf463_08, Bm_nscaf3003_091, Bm_nscaf2865_028, Bm_nscaf2868_56, Bm_nscaf3005_56, Bm_nscaf2902_244, Bm_nscaf2789_62, Bm_nscaf3033_09, Bm_nscaf2575_181, Bm_nscaf2891_057 | Bm_nscaf3035_060, Bm_nscaf2789_62 |
| 82 | Metabolism of xenobiotics by cytochrome P450 |  |  |  | Bm_nscaf3005_56, Bm_nscaf2891_057 |  |
| 83 | Mineral absorption |  |  | Bm_nscaf2855_239 |  |  |
| 84 | mRNA surveillance pathway |  |  |  | Bm_nscaf2655_005 |  |
| 85 | Natural killer cell mediated cytotoxicity |  | Bm_nscaf2136_098 |  |  |  |
| 86 | Neuroactive ligand-receptor interaction |  |  | Bm_nscaf2983_049, Bm_nscaf2674_066 | Bm_nscaf1962_13, Bm_nscaf2983_049, | Bm_nscaf463_19 |
| 87 | Neurotrophin signaling pathway |  | Bm_nscaf2136_098 |  |  |  |
| 88 | NF-kappa B signaling pathway |  |  |  | Bm_nscaf2902_300 |  |
| 89 | N-Glycan biosynthesis |  |  |  | Bm_nscaf2868_56 |  |
| 90 | Non-small cell lung cancer |  | Bm_nscaf2136_098 |  |  |  |
| 91 | Notch signaling pathway |  |  |  | Bm_nscaf2888_446 |  |
| 92 | Osteoclast differentiation |  | Bm_nscaf2136_098 |  |  |  |
| 93 | Other glycan degradation |  |  | Bm_nscaf2829_189, | Bm_nscaf463_08 |  |
| 94 | p53 signaling pathway |  |  |  | Bm_nscaf2204_083 |  |
| 95 | Pancreatic secretion | Bm_nscaf2674_064, Bm_nscaf2674_063, Bm_nscaf2983_049, Bm_nscaf2674_066 |  | Bm_nscaf2529_052, Bm_nscaf2983_049, Bm_scaffold769_2, Bm_nscaf2674_066, Bm_nscaf3035_065 | Bm_nscaf1962_13, Bm_nscaf2983_049 |  |
| 96 | Pathogenic Escherichia coli infection |  | Bm_nscaf2136_098 |  | Bm_nscaf2902_300 |  |
| 97 | Pathways in cancer |  | Bm_nscaf2136_098, Bm_nscaf3031_265 |  | Bm_nscaf3031_265 |  |
| 98 | Pentose phosphate pathway |  |  |  |  | Bm_nscaf3035_060 |
| 99 | Peroxisome |  |  |  | Bm_nscaf3026_228, Bm_nscaf2891_057 |  |
| 100 | Pertussis |  |  |  | Bm_nscaf2902_300 |  |
| 101 | Phagosome |  | Bm_nscaf3099_002 |  | Bm_nscaf2865_028, Bm_nscaf2330_003, Bm_nscaf2902_300 |  |
| 102 | Phenylalanine metabolism |  |  |  | Bm_nscaf2865_028, Bm_nscaf2902_244 |  |
| 103 | Phototransduction - fly |  | Bm_nscaf3099_002 |  | Bm_nscaf2943_045, |  |
| 104 | PPAR signaling pathway |  |  |  | Bm_nscaf2998_74, Bm_nscaf3079_28, Bm_nscaf3068_12 |  |
| 105 | Prion diseases |  |  |  | Bm_nscaf2888_446, Bm_nscaf2801_20, Bm_nscaf2801_19, Bm_scaffold944_1 |  |
| 106 | Prostate cancer |  | Bm_nscaf2136_098 |  |  |  |
| 107 | Protein digestion and absorption |  | Bm_nscaf2888_193 | Bm_nscaf2983_049, Bm_scaffold769_2, Bm_nscaf2674_066 | Bm_nscaf1962_13, Bm_nscaf2983_049 |  |
| 108 | Protein processing in endoplasmic reticulum |  | Bm_nscaf2800_73 |  | Bm_nscaf2868_56, Bm_nscaf3076_4, Bm_nscaf2801_20, Bm_nscaf2801_19, Bm_scaffold944_1 |  |
| 109 | Pyruvate metabolism |  |  |  |  | Bm_nscaf3035_060 |
| 110 | Regulation of actin cytoskeleton |  | Bm_nscaf3099_002 | Bm_nscaf2930_138 | Bm_nscaf98_59, Bm_nscaf2210_072 |  |
| 111 | Renal cell carcinoma |  | Bm_nscaf2136_098 |  |  |  |
| 112 | Retinol metabolism |  |  |  | Bm_nscaf3005_56, Bm_nscaf2891_057 |  |
| 113 | Rheumatoid arthritis |  |  |  | Bm_nscaf2902_300 |  |
| 114 | Riboflavin metabolism |  |  | Bm_nscaf2930_184 |  |  |
| 115 | Salmonella infection |  | Bm_nscaf3099_002 | Bm_nscaf2930_138 | Bm_nscaf98_59, Bm_nscaf2902_300 |  |
| 116 | Selenocompound metabolism |  |  |  |  |  |
| 117 | Serotonergic synapse |  |  |  |  | Bm_nscaf463_19 |
| 118 | Shigellosis |  | Bm_nscaf3099_002 |  |  |  |
| 119 | Small cell lung cancer |  |  |  | Bm_nscaf3031_265 |  |
| 120 | Spliceosome |  |  |  | Bm_nscaf2801_20, Bm_nscaf2801_19, Bm_scaffold944_1 |  |
| 121 | Starch and sucrose metabolism |  |  | Bm_nscaf2855_239 | Bm_nscaf2868_56 |  |
| 122 | Steroid hormone biosynthesis |  |  |  | Bm_nscaf3005_56 |  |
| 123 | T cell receptor signaling pathway |  | Bm_nscaf2136_098 |  |  |  |
| 124 | Tight junction |  | Bm_nscaf3099_002 | Bm_nscaf2930_138 | Bm_nscaf98_59 |  |
| 125 | Toll-like receptor signaling pathway |  |  |  | Bm_nscaf2902_300, |  |
| 126 | Toxoplasmosis |  | Bm_nscaf3031_265 |  | Bm_nscaf3031_265, Bm_nscaf2902_300, Bm_nscaf2801_20, Bm_nscaf2801_19, Bm_scaffold944_1 |  |
| 127 | Transcriptional misregulation in cancer |  | Bm_nscaf1108_005 |  | Bm_nscaf2839_21, Bm_nscaf2865_028, Bm_nscaf1108_005 |  |
| 128 | Tuberculosis |  |  |  | Bm_nscaf2330_003, Bm_nscaf2902_300, |  |
| 129 | Type II diabetes mellitus |  |  |  | Bm_nscaf2674_128 |  |
| 130 | Tyrosine metabolism |  |  | Bm_nscaf2930_184 |  |  |
| 131 | Ubiquinone and other terpenoid-quinone biosynthesis | |  |  | Bm_nscaf2902_244 |  |
| 132 | Vascular smooth muscle contraction |  |  | Bm_nscaf2930_138 | Bm_nscaf98_59, Bm_nscaf2210_072 |  |
| 133 | Viral myocarditis |  | Bm_nscaf3099_002 | Bm_nscaf2930_138 | Bm_nscaf98_59 |  |
| 134 | Vitamin digestion and absorption |  |  | Bm_scaffold792_1, Bm_nscaf3035_065 |  |  |
| 135 | Wnt signaling pathway |  | Bm_nscaf2655_149 |  | Bm_nscaf2655_149 |  |
|  | genes | 4 | 9 | 13 | 41 | 5 |
